# Gut Microbiota Composition Is Correlated to Grid Floor Induced Stress and Behavior in the BALB/c Mouse

**DOI:** 10.1371/journal.pone.0046231

**Published:** 2012-10-02

**Authors:** Katja Maria Bangsgaard Bendtsen, Lukasz Krych, Dorte Bratbo Sørensen, Wanyong Pang, Dennis Sandris Nielsen, Knud Josefsen, Lars H. Hansen, Søren J. Sørensen, Axel Kornerup Hansen

**Affiliations:** 1 Department of Veterinary Disease Biology, Faculty of Health and Medical Sciences, University of Copenhagen, Copenhagen, Denmark; 2 Department of Food Science, Faculty of Health and Medical Sciences, University of Copenhagen, Copenhagen, Denmark; 3 The Bartholin Institute, Rigshospitalet, Copenhagen, Denmark; 4 Department of Biology, Faculty of Science, University of Copenhagen, Copenhagen, Denmark; Cairo University, Egypt

## Abstract

Stress has profound influence on the gastro-intestinal tract, the immune system and the behavior of the animal. In this study, the correlation between gut microbiota composition determined by Denaturing Grade Gel Electrophoresis (DGGE) and tag-encoded 16S rRNA gene amplicon pyrosequencing (454/FLX) and behavior in the Tripletest (Elevated Plus Maze, Light/Dark Box, and Open Field combined), the Tail Suspension Test, and Burrowing in 28 female BALB/c mice exposed to two weeks of grid floor induced stress was investigated. Cytokine and glucose levels were measured at baseline, during and after exposure to grid floor. Stressing the mice clearly changed the cecal microbiota as determined by both DGGE and pyrosequencing. *Odoribacter, Alistipes* and an unclassified genus from the Coriobacteriaceae family increased significantly in the grid floor housed mice. Compared to baseline, the mice exposed to grid floor housing changed the amount of time spent in the Elevated Plus Maze, in the Light/Dark Box, and burrowing behavior. The grid floor housed mice had significantly longer immobility duration in the Tail Suspension Test and increased their number of immobility episodes from baseline. Significant correlations were found between GM composition and IL-1α, IFN-γ, closed arm entries of Elevated Plus Maze, total time in Elevated Plus Maze, time spent in Light/Dark Box, and time spent in the inner zone of the Open Field as well as total time in the Open Field. Significant correlations were found to the levels of Firmicutes, e.g. various species of Ruminococccaceae and Lachnospiraceae. No significant difference was found for the evaluated cytokines, except an overall decrease in levels from baseline to end. A significant lower level of blood glucose was found in the grid floor housed mice, whereas the HbA1c level was significantly higher. It is concluded that grid floor housing changes the GM composition, which seems to influence certain anxiety-related parameters.

## Introduction

From birth the mammalian gut slowly gets inhabited with a wide range of bacteria that primes the cells of the immune system during the postnatal period [1]. Throughout life, the gut microbiota (GM) remain an important factor in development of diseases, such as inflammatory bowel diseases, asthma/allergy, colon cancer, type 1 diabetes, HIV and obesity [2]. It is evident that diseases of both body and mind worsen in response to stress [3], and interest in the so-called gut-brain axis consisting of neural, immune and endocrine pathways [4] has increased, e.g. due to clinical experience with patients suffering from irritable bowel syndrome (IBS), in which a higher incidence of psychiatric illness has been acknowledged [5].

The effect of both physical and psychological stress on the gastrointestinal tract is widely recognized [6], and a recent study showed that exposure to a social stressor changed the composition of bacteria in the cecum of mature mice [7]. Moreover, it has been shown that plasma adrenocorticotropic hormone (ACTH) and corticosterone is considerably higher in germ free (GF) mice than in specific pathogen free (SPF) mice. This elevation can be reversed by reconstitution with the probiotic bacterium *Bifidobacterium infantis*, while enteropathogenic *Escherichia coli* enhance the response, and such elevated HPA (hypothalamic-pituitary-adrenal axis) response of the GF mice may partly be corrected by inoculation with SPF mouse feces at nine weeks of age, but not at 17 weeks of age [8]. 14 days of *B. infantis* administration in rats resulted in attenuation of IFNγ, TNF-α and IL-6 in plasma cytokines, which are normally elevated in response to inflammatory and stressor challenge, as well as an increase in the serotonin-precursor, tryptophan [9]. Another study concluded a significant influence of early life stress on plasma corticosterone and composition of the GM in rats [10]. Recently, it has been shown that inoculation with *Lactobacillus rhamnosus* regulates emotional behavior and central GABA receptor expression in mice [11].

Extensive literature implies that prolonged activation of pro-inflammatory cytokines play a substantial part in depression by mediating behavioral, neuroendocrine and neurochemical features [12], [13]. It has been proven by animal research and human clinical trials that the cytokines of especially the innate immune response, IL-1, IL-6 and TNFα, but also the T_H_1 cytokine IFN-γ induce “sickness behavior” characterized by the well-known symptoms of illness, such as lethargy, depression, loss of appetite and reduced grooming. Sickness behavior is believed to be part of a motivational system that changes the priorities of the organism to enhance recovery and survival [14]. A systemic cytokine response in a healthy individual is likely to originate from GM provocation of gut-associated lymphoid tissue (GALT), in which a high number of dendritic cells are present. The dendritic cell-derived cytokines IL-10 and IL-12 are generally viewed as counterparts: IL-10 promotes anti-inflammatory regulatory T-cells (T_REG_) and T_H_2-cells facilitating antibody production via B cell stimulation, whereas IL-12 facilitates a pro-inflammatory response with macrophage-activating T_H_1-cells and IFN-γ secretion [15]. Another relevant cytokine might be IL-17, as it acts on mucosal linings between the external and internal environment (intestinal mucosa, skin and respiratory tract) by recruiting monocytes and neutrophils in response to infection [16]. Thus, the balance of these cytokines in relation to GM might yield important information regarding the balance of the GM composition. Together such results emphasize the significance of early life establishment and shaping of the GM and the importance of GM composition when the individual is stressed.

Stress, anxiety and depression are generally considered interrelated phenomena; hence animal models of anxiety and depression are typically based on exposure of animals to a stressful situation. The animals are then subjected to behavioral testing to measure the behavioral and physiological reactions related to this stress situation [17]. A simple, stress-inducing environmental feature in rodents is grid floor housing, which is known to induce lasting hypertension [18], temporarily increased heart rate [19], asymmetric growth [20], [21], hippocampal changes with increased levels of neuropeptide Y and reduced levels of calcitonin gene-related peptide [22], and decreased open field activity [23].

In behavioral research in general, a variety of validated and long used behavioral tests exists for laboratory mice and other rodents, ranging from memory- and learning tasks to locomotion/activity and depression/anxiety tests. Behavioral assessment is carried out both in normal individuals as a baseline characterization parameter, for example to define phenotypes of different strains [24], and in disease models to evaluate animal welfare or disease development [25], [26], [27]. Environmental factors as well as management and study design-related factors may influence the outcome of a test. Lack of consideration for these details may lead to reduced reproducibility and higher variation.

The above described correlation between GM, cytokines and brain activity suggests that another important cause of variation to consider is the GM. Inbred mice are often chosen to reduce inter-animal variation to a minimum, but inbred mice differ significantly in their GM composition between animal units and vendors [28]. Even within inbred colonies, family relationships are still important for GM variation; pups having related mothers are more similar in GM composition than pups born from non-related mothers [29]. Previously, the complex nature of the inter-individual GM variation made it difficult to study. However, the development of culture independent methods, such as denaturing gradient gel electrophoresis (DGGE) and high throughput sequencing (HTS) based methods like 454/FLX based pyrosequencing have greatly enhanced the possibilities of studying larger bacterial communities. The DGGE method separates 16S rRNA gene derived polymerase chain reaction (PCR) products along a chemical gradient according to sequence composition [30], [31].

Based on the above, we hypothesized that stress elicited by grid floor housing induces a change in the GM of mice and that the GM is correlated to immunological and behavioral parameters.

## Results

### 1. Differences between and within Groups

#### 1.1 Blood glucose, HbA1c and cytokines

**Figure 1:**
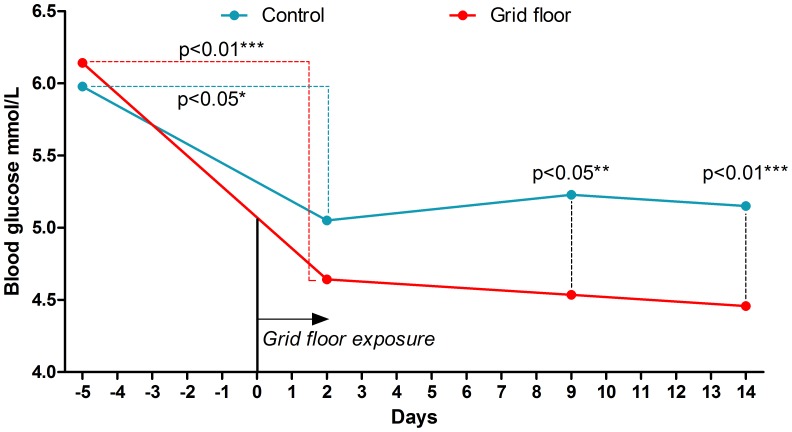
Blood glucose measurements during the study. For both groups measurements were taken at baseline, on day −5, +2, +9 of grid floor exposure for the test group and at the end of the study (mmol/L, mean and SD).

Grid floor housed mice significantly lowered their blood glucose from baseline to end (p<0.05) and ended with a significantly lower blood glucose than the control mice (p<0.01) (Table 1). When comparing the blood glucose levels within the groups from 4 days before and 2 days after grid floor exposure, the blood glucose of control mice and grid floor housed mice decreased with a significance of p<0.05 and p<0.01, respectively. Comparison between the groups on day 2 after grid floor exposure to the grid floor housed mice, revealed no significant difference. However, on day 9 there was a significant difference between the groups (p<0.05), which continued to the end of the study, where the difference was more significant (p<0.01) (Figure 1). In contrast, at the end of the experiment HbA1c values were significantly higher in the grid floor housed mice compared to the control mice (p<0.01) (Table 1).

**Table 1:** Cytokine, blood glucose and HbA1c measurements.

		Control mice				Grid floor housed mice			
*Cytokines*		pg/ml				pg/ml			
	*Time*	*Minimum*	*Median*		*Maximum*	*Minimum*	*Median*		*Maximum*
**IL-1α**	Baseline	0.00	82.30^a^		243.30	0.00	81.90^b^		475.10
	End	0.00	35.75^a^		120.06	0.00	39.15^b^		257.05
**IL-1β**	Baseline	0.00	10.37^b^		35.45	0.00	13.33^b^		39.81
	End	0.00	0.21^b^		12.68	0.00	0.00^b^		24.56
**IL-2**	Baseline	0.00	32.09^a^		70.78	0.00	33.90		71.75
	End	0.00	0.00^a^		39.72	0.00	5.29		51.66
**IL-4**	Baseline	0.00	11.89^a^		39.24	0.00	19.75^a^		49.28
	End	0.00	0.00^a^		9.57	0.00	0.00^a^		36.25
**IL-5**	Baseline	0.00	46.44^b^		99.70	0.00	47.99		176.40
	End	0.00	0.00^b^		68.93	0.00	35.67		81.76
**IL-6**	Baseline	0.00	15.57		70.76	0.00	23.56^a^		101.47
	End	0.00	5.20		191.50	0.00	1.72^a^		191.51
**IL-10**	Baseline	0.00	3.75		45.24	0.00	3.91		54.90
	End	0.00	0.00		71.37	0.00	0.00		71.37
**IL-17**	Baseline	0.00	77.10^b^		248.50	0.00	85.50^a^		251.80
	End	0.00	3.81^b^		82.30	0.00	3.81^a^		243.03
**IL-12p70**	Baseline	0.00	0.00		27.50	0.00	0.00		61.75
	End	0.00	0.00		0.87	0.00	0.00		21.61
**TNF-α**	Baseline	0.00	10.60		732.60	0.00	21.10		732.60
	End	0.00	0.00		299.70	0.00	0.00		299.70
**IFN-γ**	Baseline	0.00	2051		5187	0.00	23		5950
	End	0.00	288		4337	0.00	27		19024
***Blood glucose***		mmol/l				mmol/l			
	*Time*	*Mean*		*SD*		*Mean*		*SD*	
	Baseline	5.57		1.03		5.94^a^		1.42	
	End	5.15^b^		0.43		4.46^ab^		0.44	
***HbA1c***		mmol/mol				mmol/mol			
	End	8.86^b^		0.83		9.71^b^		0.80	

Within the same parameter and within the same group, two medians/means with the same letter are significantly different:

a p<0.05.

b p<0.01.

No significant differences were found between control mice and grid floor housed mice.

The control mice had significantly lower levels between baseline and end measurements of IL-1α (p<0.05), IL-1β (p<0.01), IL-2 (p<0.05), IL-4 (p<0.05), IL-5 (p<0.01), and IL-17 (p<0.01). In grid floor housed mice the end levels were also lower for IL-1α (p<0.01), IL-1β (p<0.01), IL-4 (p<0.05), IL-6 (p<0.05) and IL-17 (p<0.05) (Table 1). There were no significant differences in cytokines between control mice and grid floor housed mice.

#### 1.2 GM characterization

**Figure 2:**
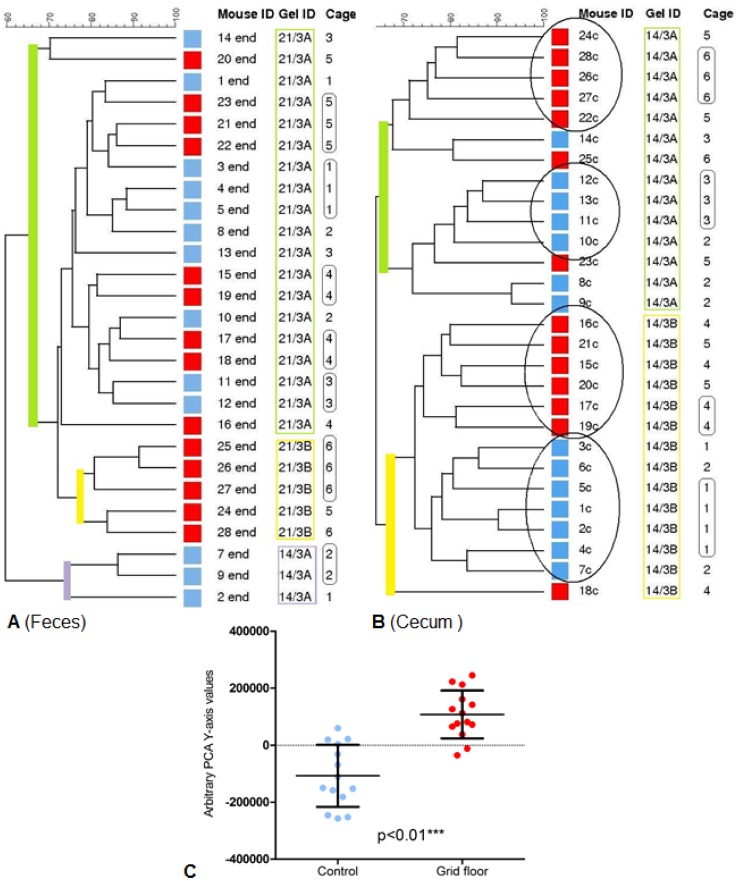
GM composition. (Blue: control mice, Red: grid floor housed mice). **A.** DGGE-profile cluster analysis similarity tree from end fecal samples. The mice clustered strongly after gel (green, yellow and purple lines) and after cage allocation (rounded squares). There was no clear clustering according to housing. Overall similarity was 59.61%±4.68. **B.** DGGE-profile cluster analysis similarity tree from end cecal samples. The mice clustered according to housing (circles) independent of gel (green and yellow) and cage allocation (rounded squares). The overall similarity was 64.07%±5.64. **C.** Scatter plot of the y-component of similarity-based cecal PCA-plot (mean and SD). Difference between control mice (−107563±108957) and grid floor housed mice (107563±84155) is significant (p<0.01).

**Figure 3:**
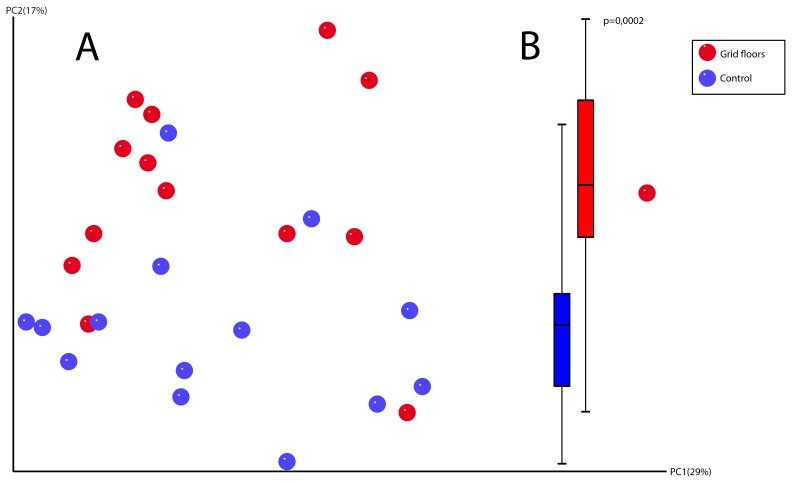
16S rRNA gene 454/FLX based pyrosequencing of cecal content. (Blue: control mice, Red: grid floor housed mice). **A.** Jackknifed replicate PCA plot. The plot is based on the phylogenetic distance matrix showing clustering of mice according to the floor type. **B.** Whisker plot of the 2^nd^ principal component values. The significant difference between the two groups observed in the DGGE-based PCA-plot is confirmed (p<0.01***).

Before the experiment (baseline) all mice clustered randomly, independent of floor type and the overall similarity of DGGE-based fecal profiles was 54%±8.13% (Figure S1). The end fecal DNA from one mouse failed to be amplified in the PCR. The overall similarity of the end fecal DGGE-profiles was 59.61%±4.68%. No clustering according to group was found (Figure 2A). The overall similarity of the cecal DNA was 64.07%±5.64%, and the mice clustered significantly according to floor type (Figure 2B), also when comparing the values from the Principal Component Analysis (PC2) (p<0.01) (Figure 2C). The differences in the cecal microbial composition between the two groups were further corroborated by tag-encoded 16S rRNA gene based pyrosequencing. The raw number of reads generated from all 28 cecal samples scored 395068. Sequences that met all requirements of the quality control (minimum length = 250 bp, quality score ≥25) yielded 214080 providing an average of 7646 sequences per sample (minimum = 5691, maximum = 10737, SD = 1198) with a mean sequence length of 448 bp (minimum = 250, maximum = 557). Taxonomic analysis disclosed 77 genera associated with either one or both groups. With an abundance threshold of 0.003% within group it was found that 36 bacterial genera were generally shared between all mice regardless of the floor type (Table S1, Figure S2 and S3). The jackknifed replicate PCA plot based on phylogenetic distance matrix showed a clear separation (p<0.01) between the two groups along PC2, describing 17% of the variance (Figure 3, A and B). Testing the rarified distance matrices (ANOSIM) proved significant difference between both categories (p<0.05). The genera *Odoribacter* (0.73% of reads in the control group *vs.* 1.12% in the grid floor group), *Alistipes* (8.44% of reads in the control group *vs.* 12.5% in the grid floor group) and an unclassified genus from the Coriobacteriaceae family (0.026% of reads in the control group vs. 0.047% in the grid floor group) were found to differ significantly in the relative distribution between the two groups (p<0.01, p<0.01, p<0.01 respectively) (Table 2). Several other genera such as *Mucispirillum* (1.1% of reads in the control group vs. 2.1% in the grid floor group) differed between the two groups, though not at a significant level (p = 0.09), but contribute to the clear separation between the two groups as seen in Figure 3.

**Table 2:** Significantly different taxa between control mice and grid floor housed mice.

Taxa	Wilcoxon ranksum test per 1000subsampled OTUtables	Control group Meanabundance	Grid floorMeanabundance	Metastats	
				p value	q value
*Bacteria;Bacteroidetes;Bacteroidia;Bacteroidales;Porphyromonadaceae;* ***Odoribacter***	1000	0.726	1.151	0.012	0.834
*Bacteria;Bacteroidetes;Bacteroidia;Bacteroidales;Rikenellaceae;* ***Alistipes***	1000	8.436	12.492	0.007	0.834
*Bacteria;Actinobacteria;Actinobacteria;Coriobacteriales;* ***Coriobacteriaceae*** *;Other*	741	0.026	0.047	0.009	0.834

Difference in bacterial relative abundance between categories verified with Wilcoxon rank sum test for 1000 subsampled OTU tables collated with results obtained with Metastats where 1000 permutations were used to calculate the p value.

#### 1.3 Behavioral tests

**Figure 4:**
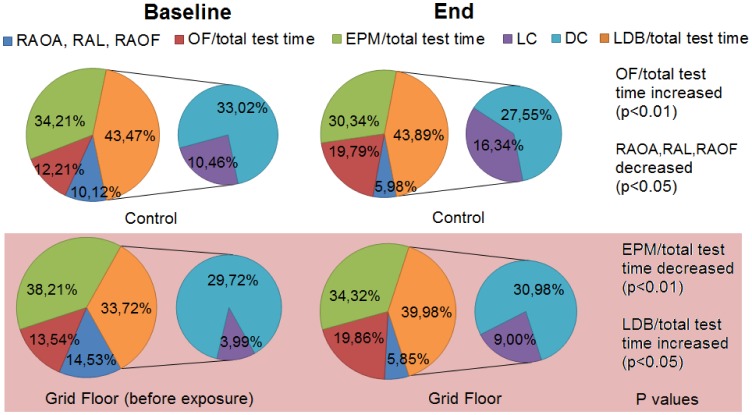
Time spent in each of the three Tripletest compartments and risk assessments. No significant differences were observed between control mice and grid floor housed mice in the overall time spent in the three compartments. Some significant changes were observed within each group. ”IZ”: time spent in IZ of total test time, “DC”: time spent in DC of total test time, “Entries CA”: number of entries into CA from OF, LDB or CA, “RAOA”: risk assessment OA, “RAOF”: risk assessment OF. “OF/total test time”: time spent in Open Field of total test time, “EPM”/total test time”: time spent in Elevated Plus Maze of total test time, “LDC/total test time”: time spent in Light (LC) or Dark Compartment (DC) of total test time, “RAOA, RAL, RAOF”: time spent risk assessing Open Arms, Light Compartment and Open Field, respectively.

**Figure 5:**
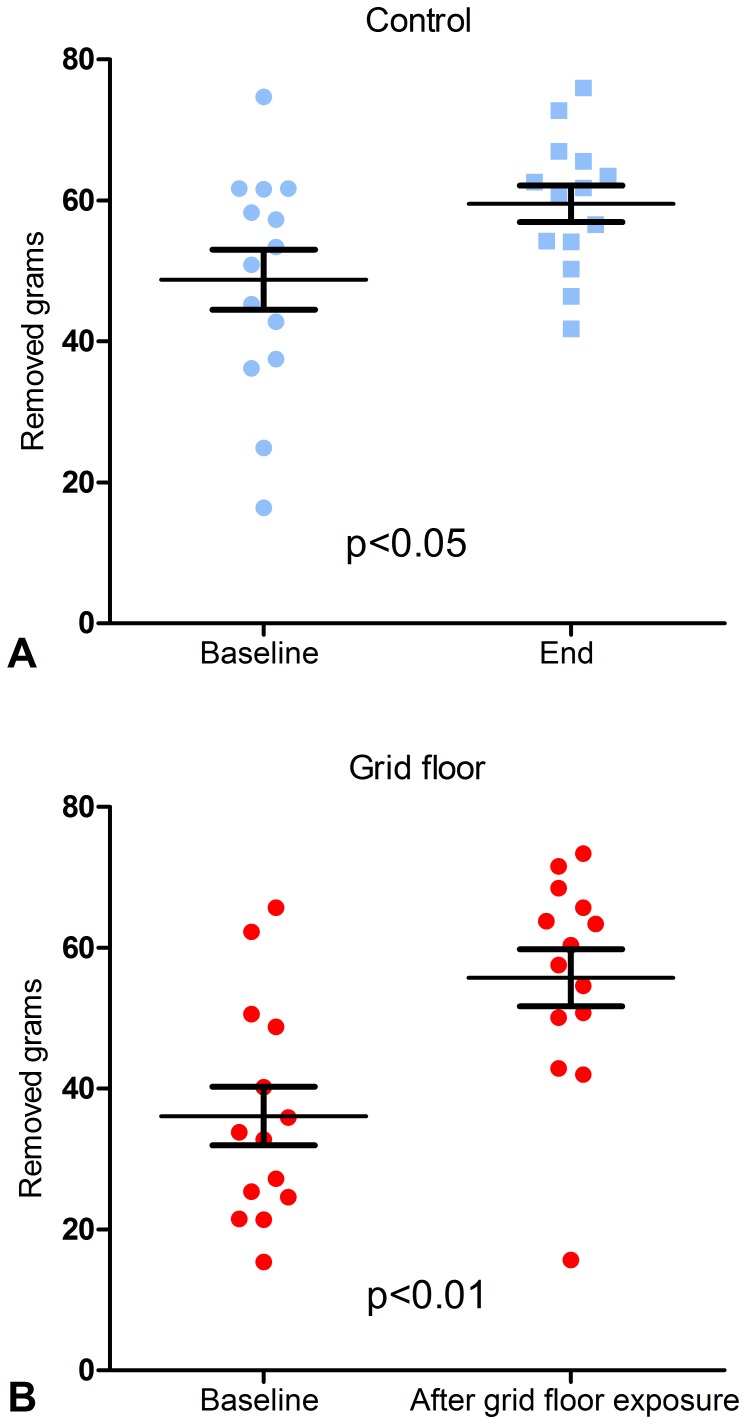
Burrowing. Scatter plots of amount for control mice (**A**) and mice housed on grid floor (**B**) (g, mean and SD). No significant difference was found between the groups. Within the groups, there was a significant difference from baseline to end, which differed between the groups with a mean of 10.77 g removed wood chips for the control mice (p<0.05) compared to 19.64 g for grid floor housed mice (p<0.01).

**Figure 6:**
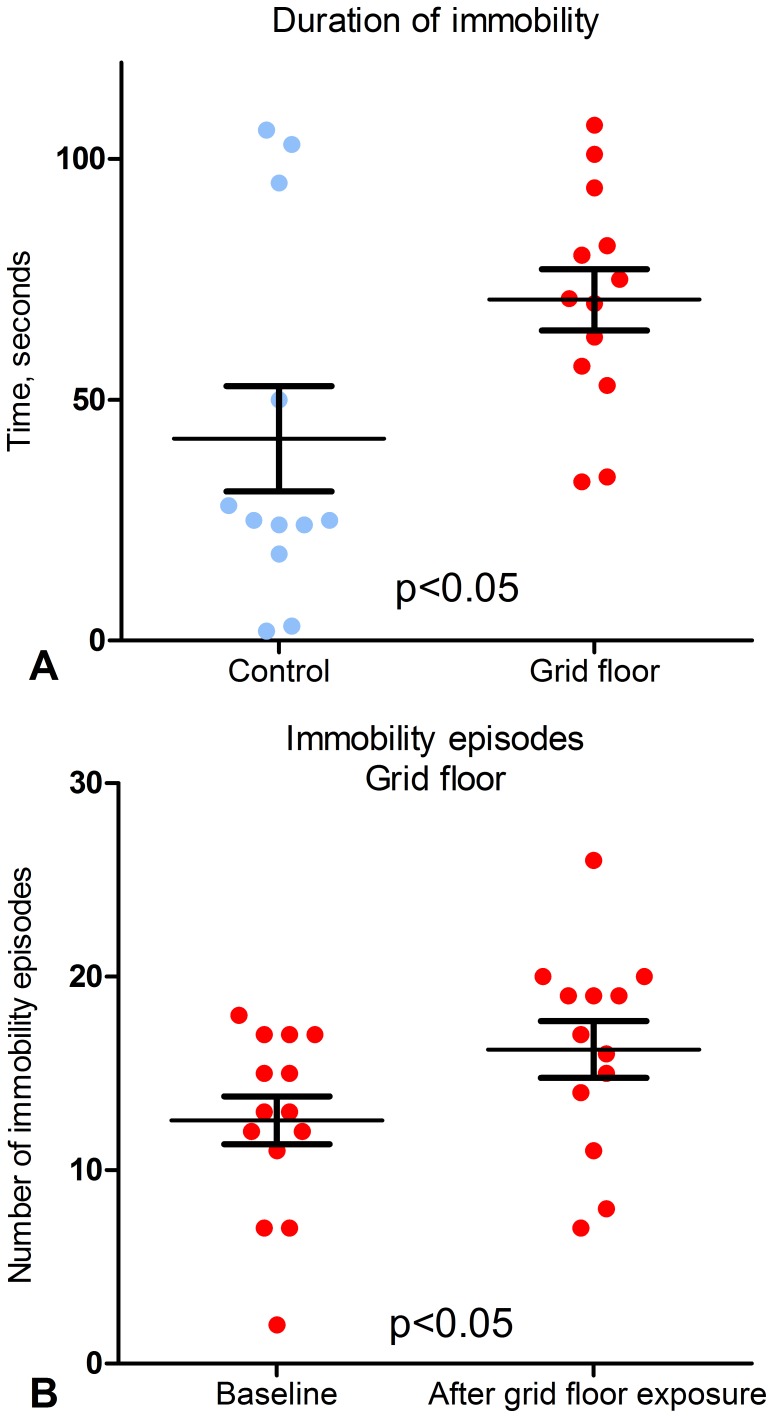
TST results. End results from three mice were not obtained, as they learned to master tail climbing. **A.** End results for immobility duration for control mice and grid floor housed mice. There was a significant difference between the groups in total duration of immobility (p<0.05) (s, mean and SD). **B.** Within-group comparison from baseline to end of grid floor housed mice. There was a significant increase in the number of immobility episodes (p<0.05) (number of episodes, mean and SD).

There were no conclusive significant differences between control mice and grid floor housed mice in the individual parameters and the overall time spent in the three compartments of the Tripletest, which was 30–38% in the EPM, 33–44% in the LDB and 12–20% in the OF. 6–15% of the time was spent risk assessing (Figure 4). Some significant changes were seen within each group. The time the control mice spent in the OF increased significantly from baseline to end of exposure, from 12% to 20% (p<0.01), and the time spent risk assessing OAs, the LC or the OF decreased significantly, from 10% to 6% (p<0.05). For grid floor housed mice, there was a significant decrease in the time spent in the EPM of total test time from 38% to 34% (p<0.01), and a significant increase in the time spent in the LDB from 33% to 40% (p<0.05). In terms of burrowing, there was a significant difference from baseline to end, which differed between the groups with a mean of 10.77 g removed woodchips for the control mice (p<0.05) compared to 19.64 g for grid floor housed mice (p<0.01) (Figure 5, A and B). In the TST the mice exposed to grid floors had significantly longer immobility duration (p<0.05) compared to the control mice, and in addition these mice increased their number of immobility episodes compared to their baseline test results (p<0.05) (Figure 6, A and B).

### 2. Correlation between GM, Plasma Parameters and Behavioral Parameters

There was no correlation between any of the clinical parameters and the baseline fecal DGGE (cut-off value: 70%; 9 clusters). In the end fecal parameters, correlation of Entries CA and time spent in LDB/total test time with fecal DGGE-cluster groups (Cut-off 77%; 9 clusters) was significant (p<0.05 for both). For cecum DGGE-cluster groups (Cut-off 76%; 7 clusters) a significant correlation with time spent in EPM/total test time (p<0.05), IL-1α and IFN-γ (p<0.01 for both) was found. Choosing a smaller cut-off value of 68% reduced the number of clusters to three in the cecum similarity tree (see Figure 2B). For these clusters in addition to the significant correlation with time spent in EPM/total test time, another significant correlation was found for time spent in OF/total time (p<0.01) as well as time spent in the IZ/OF (p<0.01), while these clusters showed no significant correlation with the cytokines IL-1α and IFN-γ. This was confirmed by a significant correlation between the PC2 values of the cecum PCA plot and time spent in EPM/total test time (p<0.01) of grid floor housed mice. In the tag-encoded 16S rRNA gene based pyrosequencing data of the caecal flora of the grid floor group significant correlations were found to the levels of Firmicutes, i.e. the level of various species of Ruminococccaceae correlated to entries CA (p<0.05), time spent in DC (p<0.05) and to IL-2 (p<0.05), while the level of Lachnospiraceae correlated to RAOA (p<0.05). There was also a significant correlation between the level of Bacterioides and the number of immobility episodes in the TST in the control group (p<0.05) (Table 3).

**Table 3:** Bacterial levels of correlation between pyrosequencing data from cecal flora and parameters.

Parameter	Taxa	MINE per 1000subsampledOTU tables	AverageMICvalue	p	pos/neg
	**Control group**				
“immobility”	*Bacteroidetes;Bacteroidia;Bacteroidales;Bacteroidaceae;* ***Bacteroides***	876	0.617	<0.05**	**+**
	**Grid floor group**				
“RAOA”	*Firmicutes;Clostridia;Clostridiales;* ***Lachnospiraceae*** *;Other*	993	0.677	<0.01***	**+**
“entriesCA”	*Firmicutes;Clostridia;Clostridiales;Ruminococcaceae;* ***Butyricicoccus***	813	1.000	<0.01***	**−**
“entriesCA”	*Firmicutes;Clostridia;Clostridiales;Ruminococcaceae;* ***Oscillibacter***	861	0.750	<0.01***	**+**
“DC”	*Firmicutes;Clostridia;Clostridiales;* ***Ruminococcaceae*** *;Other*	957	0.689	<0.01***	**+**
“IL2”	*Firmicutes;Clostridia;Clostridiales;* ***Ruminococcaceae*** *;Other*	957	0.689	<0.01***	**−**

“Immobility”: Immobility episodes in the Tail Suspension Test, “RAOA”: Risk Assessment of Open Arms in the Elevated Plus Maze, “entriesCA”: Entries into the closed arms of the Elevated Plus Maze, “DC”: Dark Compartment of the Light/Dark Box, “IL2”: Interleukin-2.

## Discussion

Stressing BALB/c mice by housing them on grid floor induced a change in the cecal microbiota composition. It is interesting that *Alistipes* spp. were found to be more abundant in the grid floor housed mice, as this bacterium is known to be correlated to pain in pediatric patients with Irritable Bowel Syndrome (IBS) [31], but it is not possible from our study to point at specific bacteria relating to specific behavioral effects. IBS seems to be associated with the composition of the microbiome [32], and in humans, early-life stress as well as stress episodes in adult life are known as important risk factors in the development of IBS [33]. It is therefore interesting that it seemed to be the activity in the elevated plus maze, i.e. the total time spent and the entries into the closed arms, that correlated to GM composition of cecum and feces, respectively, and that the grid floor housed mice decreased the time they spent in the EPM, as this is the classical and most popular rodent anxiety test [25], [34]. Germ free mice have reduced anxiety-like behavior in this test [35], and they preserve this behavior when reconstituted with a GM [36]. Consequently there seems to be an age window during which the GM impact on the gut-brain axis is critical. During such a window, changes in GM have been found to have lasting effect on the host [37] and have also been observed from animal studies on other diseases under the impact of the GM, e.g. type 1 diabetes [38]. As the time spent in the EPM entails activities of both open and closed arms, this decrease in time for the grid floor housed mice should in our study be interpreted with caution, but it definitely means that more time was spent away from this compartment of the Tripletest. Incorporating the EPM into the Tripletest as the middle ‘transit’ compartment may make the CA a better measure for activity rather than anxiety. Compared to presenting the mouse to one test at a time or to only one test, the exploration possibility of the combined test is greatly enhanced [39], and it eliminates confounding factors like previous test experience, inter-test intervals, time of day-variance and handling between tests [40]. For grid floor housed mice, there was a significant increase in the time spent in the LDB, and also, there was a significant correlation to the end fecal GM. The LDB box is also a classical expression of anxiety-like behavior in mice, and usually decreased time in the LC is viewed as anxiety-related [41]. In our study is seems that the grid floor housed group spend the same amount of time in the dark compartment before and after grid floor exposure, whereas the control group spend less time in the dark compartment pointing to more exploratory behavior. However, no conclusive significance was found in these data. Another classical expression of anxiety is time spent in the IZ of the OF arena, e.g. away from the open space [42], for which there also was a significant correlation to the cecum microbiota in our study.

The phylum Coriobacteriaceae, which increased in the grid floor housed mice, has previously been shown to decrease when High Density Lipids (HDL) increased in a hamster model [43]. A specific species within Coriobacteriaceae, *Mt1B8*, is known to be able to degrade the isoflavones daidzein and genistein to equol [44], which seems to be the compound responsible for some of the metabolic effects of isoflavones [45]. Isoflavone feeding is linked to a lowering of insulin resistance and HbA1c [46]. The grid floor housed mice had lower blood glucose than the control mice, though stress causes an increase in blood glucose [47]. Interestingly, the HbA1c was significantly higher in the grid floor group. The blood glucose is sensitive to daily fluctuations in normal homeostasis, and therefore the long-term blood glucose messenger HbA1c is a more correct evaluation of blood glucose. Because the equipment was newly acquired in the laboratory in the end of the experiment, no HbA1c baseline measurement exists to compare with. Nevertheless, the higher level of HbA1c in the grid floor housed mice is concomitant with an overall stressful effect of the grid floors. The blood glucose levels were measured on different time points during the study. Between 2 and 9 days after application of the grid floors to the test group, a clear difference between the groups was seen. The overall fall in blood glucose in both groups from day −5 to +2 from grid floor exposure to the test group is possibly a result of decreased food intake. The baseline behavioral tests were performed on the two days prior to grid floor application, and the commotion of being disturbed in the cage, transported to another room and tested is naturally both a stress factor and a disturbance of the daily cage rhythms. Stress causes decreased food intake in laboratory rodents [48], so the low blood glucose level in both groups is not surprising. However, the statistical significance is more pronounced in the grid floor housed mice than in the control group, and though highly speculative, this suggests that the grid floor is an add-on stressor.

It is difficult to interpret these results, but it could be hypothesized that changes in the GM composition can be seen as part of a long term compensation for the stress.

The species *Odoribacter*, which increased in the grid floor housed mice compared to the control group, is a normal member of the human GM [49], [50], [51], but have seemingly not been related to any clinical features in man or animals.

In mice, there only seem to be a minor correlation between the GM composition of cecum and feces [52]. The fact that the behavioral data primarily correlated to the cecal microbiota may be explained by the higher relevance of the cecum contents in relation to the lymphatic tissues of the ileal Peyer patches. It is indeed reasonable to assume that the effect may be elicited by a gastro-intestinal tract immune activation [5], as we did find a positive correlation between the cecal microbiota composition and the pro-inflammatory serum cytokines IL-1α and IFN-γ. These cytokines are representatives of innate and adaptive immunity, respectively, and this association is thus not surprising. Pro-inflammatory cytokines, such as IL-1α, are known to be positively correlated with depression-like behavior and stress, inducing “sickness behavior” [12], and this is interesting, as grid floor exposure was also correlated with these same cecal cluster groups. Although stress in more popular terms is viewed as an immunosuppressive condition, depending on the type and duration of the stressor and the immunological parameter in question, stress responses can also enhance or have no effect on immunological parameters [53]. That it primarily seems to be Ruminococcaceae spp. that correlates to behavior is difficult to interpret, as little is known about the impact of these species. One of the human enterotypes recently defined is dominated by Ruminococcaceae [54], and it is known from patients with hepatic encephalopathy that the levels of Ruminococcaceae correlate negatively to inflammation [55].

Although it could be speculated that the grid floor effect on the GM was due to the reduced contact with the microbial environment, it is unlikely as laboratory mice and other rodents practice coprophagy directly from the anus as a part of nutritional requirements [56]. The differences we found in grid floor housed mice supports the hypothesis of grid floors being stressful, as it is widely accepted that stress can induce disturbances in the GM leading to gastro-intestinal disease [6]. In fact a recent article showed that stressor exposure of mice significantly changed the community structure of the microbiota in the cecum [7]. This may not necessarily be measurable in feces as GM communities at the various gut sites and in feces are significantly different in both humans and mice [52], [57]–[62]. The TST seemed to be the most sensitive test to a possible grid floor effect, as grid floor housed mice had a longer total duration of immobility compared to the control mice. Also, within the group of grid floor housed mice, there was an increase in the number of immobility episodes from baseline to end. Furthermore, ANOVA analysis confirmed that grid floor exposure was an independent factor for the number of immobility episodes. The TST is widely used in screening of anti-depressants in animal models [63] having been stressed into a depression-like state by the use of chronic mild stress, resulting in additional and longer immobility episodes [26]. The fact that the same effects were generated in this experiment, where the stress factor was grid floor exposure for two weeks, implies that the use of grid floor is a sufficient stressor for inducing a depression-like behavioral state in the TST.

Our studies indicate that changes in GM composition induced by grid floor housing may interfere with behavioral testing, as well as with studies of a range of inflammatory diseases [64]. Grid floor housing is not commonly accepted as routine housing in European regulations [65], although still applied in other parts of the world. This housing option may not only have obvious animal welfare concerns relating to stress, but should also be regarded as having a strong impact on a range of animal models, which makes it doubtful also from a scientific point of view.

In conclusion, stressing mice by housing them on grid floor changes the GM composition and influences certain anxiety-related parameters. There is a correlation between certain elements of such anxiety-related behavior and some undefined elements of the GM; an effect which may be elicited by pro-inflammatory cytokines.

## Materials and Methods

### 1. Mice

The experiment was conducted in accordance with the Council of Europe Convention European Treaty Series (ETS) 123 on the Protection of Vertebrate Animals used for Experimental and Other Scientific Purposes, and the Danish Animal Experimentation Act (LBK 1306 from 23/11/2007). The study was approved by the Animal Experiments Inspectorate under the Ministry of Justice in Denmark (License number: 2007-561-1434 C8). All efforts were made to minimize the number of animals used and to minimize suffering.

28 female BALB/cAnNBomTac mice aged five weeks at arrival were randomly allocated to six standard cages (type 1290, Techniplast, Italy) with 4–5 mice in each cage. Group 1 (n = 14) was the control group, and group 2 (n = 14) was exposed to grid floors (raised bottom grids, Techniplast, Italy) from day 26 until the end of the study (Table 4). The mice were housed under a 7 am–7 pm light/dark cycle and 440 lx light intensity in front of the cage rack. Relative humidity was 55% +/−10%, and the temperature was 20–24°C. The mice were fed a standard Altromin 1324 diet (Altromin, Germany) *ad libitum* and tap water in bottles. They were housed on aspen bedding (Tapvei, Estonia) with supplement of Enviro-dri and Alpha-Nest nesting material (SSP, USA), Shepherd’s Shacks (regular, SSP, USA), and an aspen chew block (Tapvei, Estonia).

**Table 4:** Timeframe with number of days, age of the mice and tests performed.

Day	Age	Mice^a^	Tests/Activity
**0**	5 w	All	Arrival, randomization and ID
**0–16**		All	Acclimatization period
**17**	7 w	Group 1 A (n = 7) and Group 2 A (n = 7)	Blood glucose, blood and fecal samples
**18**		Group 1 B (n = 7) and Group 2 B (n = 7)	Blood glucose, blood and fecal samples
**21**	8 w	All	Blood glucose
**24**		Group 1 A (n = 7) and Group 2 A (n = 7)	Tripletest, Burrowing, Tail Suspension Test
**25**		Group 1 B (n = 7) and Group 2 B (n = 7)	Tripletest, Burrowing, Tail Suspension Test
**26**		Group 2	*Grid floor for the remaining time*
**28**	9 w	All	Blood glucose
**35**	10 w	All	Blood glucose
**38**		Group 1 A (n = 7) and Group 2 A (n = 7)	Tripletest, Burrowing, Tail Suspension Test
**39**		Group 1 B (n = 7) and Group 2 B (n = 7)	Tripletest, Burrowing, Tail Suspension Test
**40**		Group 1 A (n = 7) and Group 2 A (n = 7)	Blood glucose, Hb1Ac, blood and fecal samples. Euthanization and cecum removal.
**41**		Group 1 B (n = 7) and Group 2 B (n = 7)	Blood glucose, Hb1Ac, blood and fecal samples. Euthanization and cecum removal.

a Group 1 was the control group housed on solid floors and group 2 was exposed to grid floors for 15 (group 2 A) and 16 (group 2 B) days, respectively. This one-day window was necessary for adequate time to euthanize and take out samples from the mice. Half of each group was tested on the same day, and half of the mice in a cage were tested on the same day to avoid day-to-day and cage biases.

### 2. Behavioral Tests

All behavioral tests were performed twice on both groups; as a baseline measure prior to grid floor housing of the test group and at the end of the study (Table 4).

**Figure 7:**
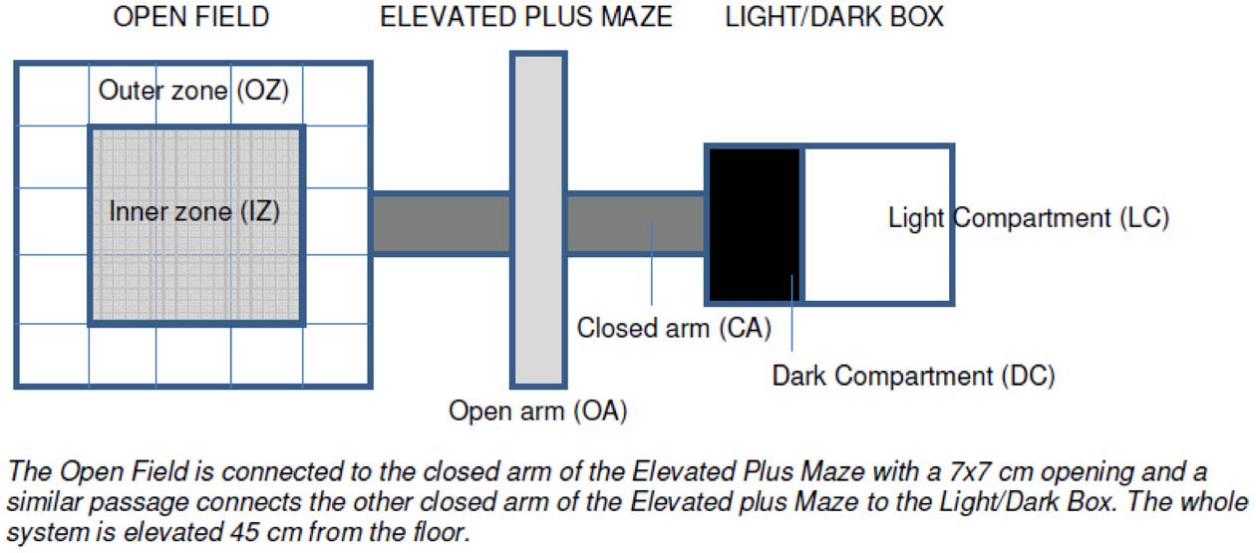
Overview of Tripletest construction. The Open Field is connected to the closed arm of the Elevated Plus Maze with a 7×7 cm opening and a similar passage connects the other closed arm of the Elevated Plus Maze to the Light/Dark Box. The whole system is elevated 45 cm from the floor.

***“The Tripletest”*** combining the Open Field (OF), the Elevated Plus Maze (EPM), and the Light/Dark Box (LDB) [40], [41] was used (Table 5 and Figure 7) with light intensities over the apparatus of 55.8 lx (middle of the EPM), 101.7 lx (middle of OF), 109.3 lx (light compartment, LC), and 1.2 lx (dark compartment, DC). A mouse was placed in the center of the OF and observed for 15 minutes as it explored the apparatus. Two video cameras recorded the test; one was placed above the OF, and the other covered both the EPM and the LDB. The behavioral parameters were obtained manually by watching the two videos simultaneously to ensure continuity.

**Table 5:** Construction details of the Tripletest.

Compartment	Overall dimensions	Other measures	Colour and adjustments
**Tripletest system in general**	140.5 cm long	Lifted 45 cm from the floor Openings between compartments: 7×7 cm	–
**Open Field (OF)**	24×47.5×47.5 cm	–	White, marked with 25 squares
**Elevated Plus Maze (EPM)**	50.5×50.5 cm	Arms: 21 cm longClosed arm walls: 30 cm	Grey, marked with 3 squares on open arms
**Light/Dark Compartment (LDB)**		Dark compartment: 22×21×14.5	Black, and closed with a lid
		Light compartment: 22×21×23	White

From the OF, time spent and squares travelled in outer (OZ) and inner zone (IZ) was noted. From the EPM, time spent in and number of entries (all four paws crossed into an arm) into open arms (OA) and closed arms (CA) and the total time spent in the whole compartment was noted. Risk assessment (RA) was assumed when two paws were placed into an open arm and then retracted. The frequency of risk assessments was noted. From the LDB, time spent in each compartment (LC and DC), and number of entries into the light compartment (LC) was noted, as well as the number of risk assessments of the LC. The overall number of event codes for each mouse was also noted. The apparatus was cleaned if necessary between each mouse, but always if urination had occurred. From the collected parameters, the following were selected for statistical analysis: Entries into CA and OA in the EPM as a measure of anxiety/activity, time spent in DC, in LC and in IZ as a measure of anxiety, risk assessments, and overall time distribution between LDB, EPM and OF. The number of events for each mouse (the number of written event codes) was also tested.

***Burrowing*** applied as a measure of species-specific activity in mice [66]. The mice were placed individually in cages similar to the home cages containing a burrowing tube filled with exactly 80 g of wood chips. The cages were covered with paper diapers for limitation of stressful distractions. After two hours the remaining contents of the tube were weighed and the mice returned to their home cages.

***The Tail Suspension Test*** (TST) [26] was applied as a measure of depression-like behavior (behavioral despair). A black wooden box of plywood measuring 40×40×30 cm was used for the test. A metal hook was placed in the roof for suspension. Light intensity inside the TST box was 63.5 lx. A paper clip was attached one cm. from the tip of the tail of the mouse with Millipore medic tape and then connected to the hook in the roof. Mice were tested individually for six minutes, and the test was videotaped. Mice were considered immobile only when hanging passively and completely motionless. If a mouse engaged in tail climbing it was eliminated from the test. Number of immobility episodes and total duration of immobility were recorded manually by watching the videos.

### 3. Physiological Parameters

#### 3.1 Blood glucose and HbA1c

The blood glucose was measured with a glucometer (FreeStyle Lite® Blood Glucose Monitoring System, Abbott) between 8–10 am each time by lateral tail vein puncture. HbA1c (glycated hemoglobin) was measured at the end of the experimental period on a DCA Vantage machine (Siemens, Germany) according to manufacturer’s protocol.

#### 3.2 Cytokines and C-reactive protein

Survival blood samples were obtained from the mice by cheek puncture. Non-survival blood was sampled by infra-orbital sinus puncture under anesthesia with fentanyl, fluanisone and midazolam, i.e. 1∶1 *Hypnorm/Dormicum* mixture: 0.315 mg/ml fentanyl +10 mg/ml fluanisone (VetPharma, UK) and 5 mg/ml midazolam (Roche, Denmark) after which the mice were immediately euthanized by cervical dislocation. Blood samples were stabilized in EDTA-coated micro tubes (Eppendorf, Germany), placed on wet ice during collection and later centrifuged at 4000 rpm for 6 minutes. Plasma was stored at −80°C before the cytokines IL-1α, IL-1β, IL-2, IL-4, IL-6, IL-10, IL-12, IL-17,TNF-α and INF-γ were measured on a BD FacsCanto Flow Cytometer (BD Biosciences, Denmark) using a Mouse Th1/Th2 10plex FlowCytomix Kit with supplemental simplex beads (Bender MedSystems; products no. BMS820FF, BMS86004FF and BMS86002FF) according to manufacturer’s protocol with plasma and reagents downscaled by 50%. The data processing was performed using the FlowCytomix™ Pro 2.3 Software (Bender MedSystems). Acute-phase protein CRP was measured by ELISA (DRG Diagnostics, Germany; CRP (mouse) ELISA, product no. EIA-4823) according to the manufacturer’s protocol, and results were read on a Sunrise Micro plate Absorbance Reader (Tecan, Switzerland).

#### 3.3 GM characterization

DNA extraction: One or two fecal pellets from each mouse were collected by voluntary defecation into an autoclaved microtube (Eppendorf, Germany) placed on wet ice during collection, and then stored at −80°C. After euthanasia, 0.5 cm of the cecum with contents was aseptically removed and stored in microtubes at −80°C. Bacterial DNA was extracted following the protocol for pathogen detection from Qiagen (QIAamp DNA Stool Mini Kit 51504). The cecum samples were homogenized in a FastPrep FP120 Cell Disrupter (Qbiogene, MP Biomedicals, France) at 6 m/sec for minimum 45 seconds. The quality and quantity of the DNA were evaluated on a NanoDrop 1000 Spectrophotometer (Thermo Scientific, USA). Extracted DNA was stored at −40°C.

DGGE: The V3 region of the 16S rRNA gene was amplified by using a universal primer set, namely PRBA338f and PRUN518r (Eurofins MWG Operon, Ebensburg, Germany). All reactions were carried out in a 50 µl volume containing 5 µl 10× HotMaster Taq Buffer (5 Prime, Germany), 12 µl dNTP (1.25 mM; Bioline, Germany), 1 µl forward primer, 1 µl reverse primer, 1 µl bovine serum albumin (5 µl/ml, Sigma-Aldrich, Denmark), 0.25 µl (1.25 U) HotMaster Taq DNA Polymerase (5 Prime), MilliQ water (to the volume of 50 µl), 3 µl caecal DNA or 5 µl fecal DNA (approximately 50 ng DNA). The PCR was conducted on a GeneAmp PCR System 9700 (Applied Biosystems, Life Technologies, USA), with initial denaturation at 95°C for 5 minutes, then 30 cycles of denaturation at 95°C for 30 seconds, annealing at 60°C for 30 seconds and extension at 72°C for 40 seconds followed by a final elongation step at 72°C for 10 minutes. The PCR products, sized approximately 230 base pairs, were checked by electrophoresis on a 2% agarose gel at 100–120 V for 20 minutes. The gel was stained with Ethidium Bromide for 10 minutes and then visualized (AlphaImager HP, Cell Biosciences, USA). The PCR products were stored at −20°C. Denaturing Grade Gel Electrophoresis (DGGE) was performed on an INGENYphorU-2 (Ingeny International BV) according to manufacturer’s protocol. The acrylamide concentration in the gel was 9% and the denaturation gradient was 30 to 65%. 20 µl of the PCR product mixed with 3.75 µl 6× DNA loading dye (Fermentas, Thermo Fisher Scientific, USA) was loaded into the wells of the stacking gel. A standard mixture, consisting of one mouse with a wide range of bands and three random mice, was loaded for every four to six samples. Electrophoresis was performed in 17 l MilliQ water with 170 ml 50× TAE buffer added, at 60°C for 16 hours at 120 V. The gels were stained with 25 µl SYBR Gold (Invitrogen, USA) dissolved in 250 ml buffer solution taken from the electrophoresis tank, for one hour and photographed with UV transillumination (EDAS, Eastman Kodak, USA).

Tag-encoded pyrosequencing: Sequencing of the16S rRNA gene V4 region amplicons from cecal samples was carried out in the National High-throughput DNA Sequencing Centre, Denmark, according to manufacturer’s instructions (Roche) and manual previously described by Hansen et al. [38].

### 4. Data Management and Statistics

#### 4.1 Differences between and within groups

Parameters of the Tripletest and Burrowing were tested statistically with 95% confidence interval in GraphPad Prism 5.02 (GraphPad Software, USA), and a cut-off of 800 seconds test time was used for the Tripletest. All data were tested with D’Agostino and Pearson omnibus normality test. For initial analysis, normally distributed data were tested with one-way ANOVA with Bonferroni Post Test, and for confirmation of significance paired or unpaired students t-test was used. Non-parametric and small sample size data were initially tested with Kruskal-Wallis with Dunns Post Test, and significance was confirmed with Wilcoxon matched pairs test or Mann-Whitney test. The number and position of bands of DGGE were defined manually and managed with BioNumerics 4.5 (Applied Maths, Belgium). Dendrograms were obtained by performing similarity analysis using the Dice similarity coefficient (band position tolerance and optimization of 1%) and the un-weighted pair-group method with arithmetic averages clustering algorithm (UPGMA). Principal Component Analysis was performed to visualize the DGGE data in 3-dimensional plots, and to extract PC1, PC2 and PC3 component values to compare statistically with behavioral and physiological parameters.

#### 4.2 Sequencing data analysis

The 454 pyrosequencing data was analyzed using the software package Quantitative Insights Into Microbial Ecology (QIIME, http://www.qiime.org/index). The quality control step followed by sequences sorting and trimming was set to keep all sequences with the average quality score ≥25 and a minimum length of 250 bp. The improvement of imprecise signals for long homopolymers (denoising) was conducted with the denoise_wrapper.py script. The putative chimeric sequences were purged using ChimeraSlayer [67]. Preprocessed sequences were further clustered at 97% relatedness using UCLUST (http://www.drive5.com/usearch/). The representative sequences from each cluster were aligned with pyNAST [68] and subjected to the Ribosomal Database Project (RDP)-based 16S rRNA annotation.

The Jackknifed replicate PCA plot was generated by Jackknifed Beta Diversity workflow with the -e value (number of sequences taken for each jackknifed subset) equal to the 80% of the sequence number within the most indigent sample. The difference between the two categories was tested with the ANOSIM test based on ten rarefied, weighted UniFrac distance matrices (average p value <0.05). Testing for significantly different Operational Taxonomic Units (OTUs) between the control and the grid floor groups was performed using Metastats (http://metastats.cbcb.umd.edu/). The number of permutations used to calculate the p value (significance threshold = 0.05, false discovery rate threshold = 0.5) was set to 1000. Additionally a thousand rarified OTU tables with normalized number of reads (5500 for each sample) were generated. Taxonomic groups in each OTU table were summarized at the genus level and differences in the bacterial relative distribution between the two groups were tested using the Wilcoxon rank sum test. All taxa that reached significant difference (p≤0.05) in each rarified OTU table were collated and compared with the results from Metastats (Table S1). The subsampled OTU tables were made using multiple_rarefaction_even_depth.py and summarize_taxa.py scripts while the group’s comparisons with Matlab in-house script (http://www.mathworks.com).

#### 4.3 Correlation between GM and other parameters

Cluster groups allocating the mice in groups according to cluster similarity were achieved by defining a cut-off value on the dendrograms at a point where visual clustering could be observed. This was done for both DGGE and pyrosequencing data. It was analyzed in Minitab 16 (Minitab Inc., USA), by analysis of variance (ANOVA) using a general linear model with the model determined as [“Cluster” Treatment “Cluster”] with cage number and placement as covariates. PC1, PC2 and PC3 of the PCA plots were tested group wise for normal distribution in Anderson Darling test. A combined model for the axes PC1, PC2 and PC3 (separately for DGGE and pyrosequencing) was used to perform linear regressions to see if the combination of PC1, PC2 and PC3 could be used as predictors of clinical or immunological parameters (Minitab Inc., Coventry, UK). The maximal information coefficient measure (MIC) was exploited to assess the relationship between pyrosequencing based bacteria abundance and clinical parameters. A 1000 subsampled OTU tables were generated (5500 sequences per sample) and correlated individually with the reference parameters using MINE (http://www.exploredata.net) in order to validate how many times, within all rarefied tables, a given relationship will remain significant (p<0.05).

## Supporting Information

Figure S1 **DGGE-profile cluster analysis similarity tree.** From baseline fecal samples (light blue: control group; dark blue: test group). The mice clustered strongly after gel (green and yellow) and after cage allocation (rounded squares). There was no cluster separation of the groups. Overall similarity was 54%±8.13%.
(TIF)

Figure S2 **Relative distribution of genera.** Bar chart presenting the relative distribution (% of total) of all 77 genera found in the cecum within the two groups of mice as determined by 16S rRNA gene 454/FLX based pyrosequencing. See Table S1 for colour legends.
(TIF)

Figure S3 **Shared genera.** Shared genera between the control group (red) and grid floor housed mice (blue) as determined by 16S rRNA gene 454/FLX based pyrosequencing. The circle nodes denote the 36 genera that reached the abundance threshold of 0.003% within each group. As seen all genera present above the threshold value are represented in both groups of mice.
(TIF)

Table S1 **Relative distribution (% of total) cecum microbiota as determined by 16S rRNA gene 454/FLX based pyrosequencing for each mouse.** The last four columns include the mean values for each genus in the groups and the significantly different taxa between categories analysed with Metastats and the Wilcoxon rank sum test performed for 1000 subsampled OTU tables. Colour codes refer to Figure S1.
(PDF)
