# Supplementary figures and images for: Gut Microbiota Composition Is Correlated to Grid Floor Induced Stress and Behavior in the BALB/c Mouse
Source: PLoS One. 2012 Oct 2;7(10):e46231. doi: 10.1371/journal.pone.0046231 (PMC3462757; doi:10.1371/journal.pone.0046231)

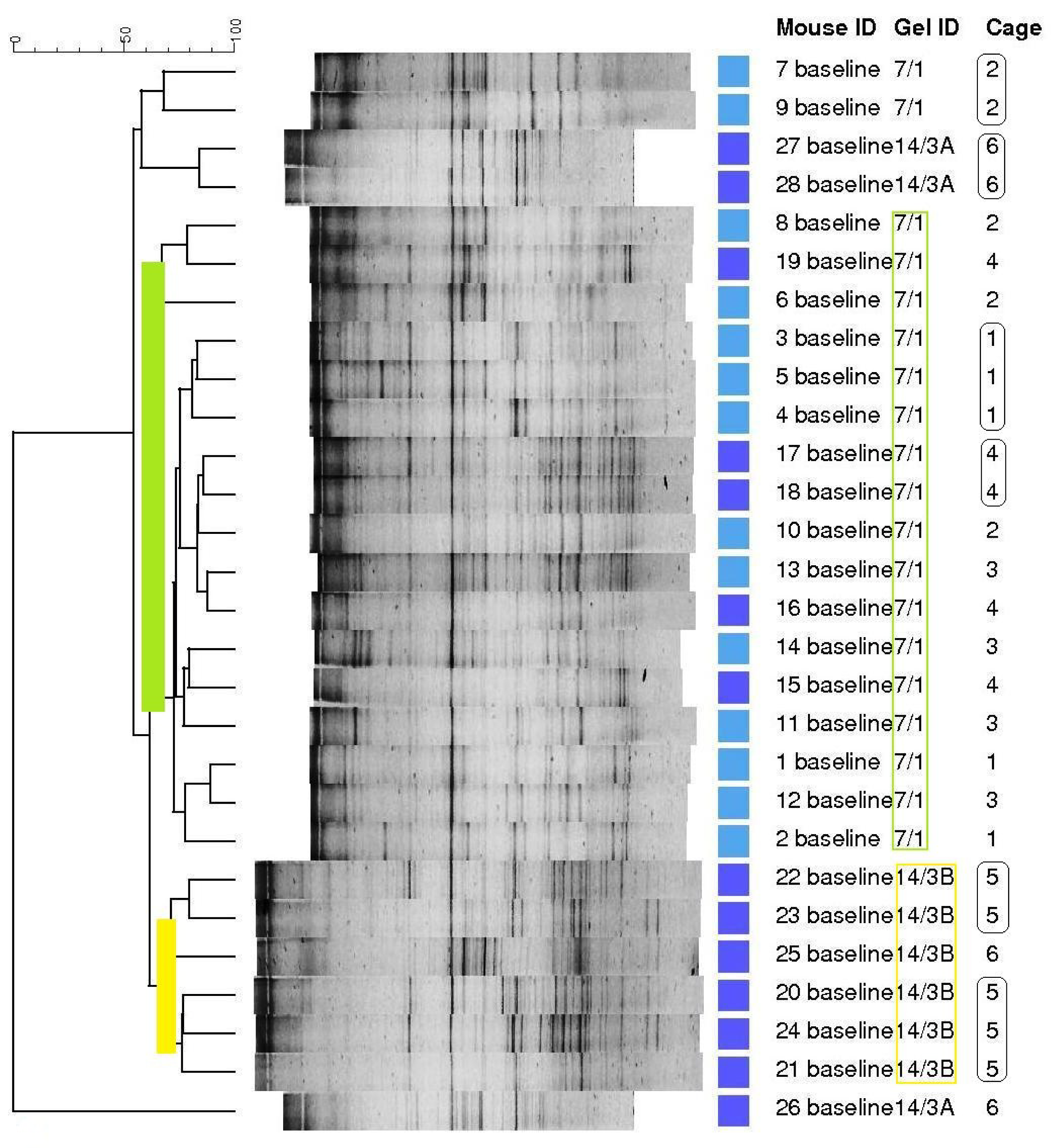

Supplement: Figure S1 — DGGE-profile cluster analysis similarity tree. From baseline fecal samples (light blue: control group; dark blue: test group). The mice clustered strongly after gel (green and yellow) and after cage allocation (rounded squares). There was no cluster separation of the groups. Overall similarity was 54%±8.13%. (TIF) [file pone.0046231.s001.tif]

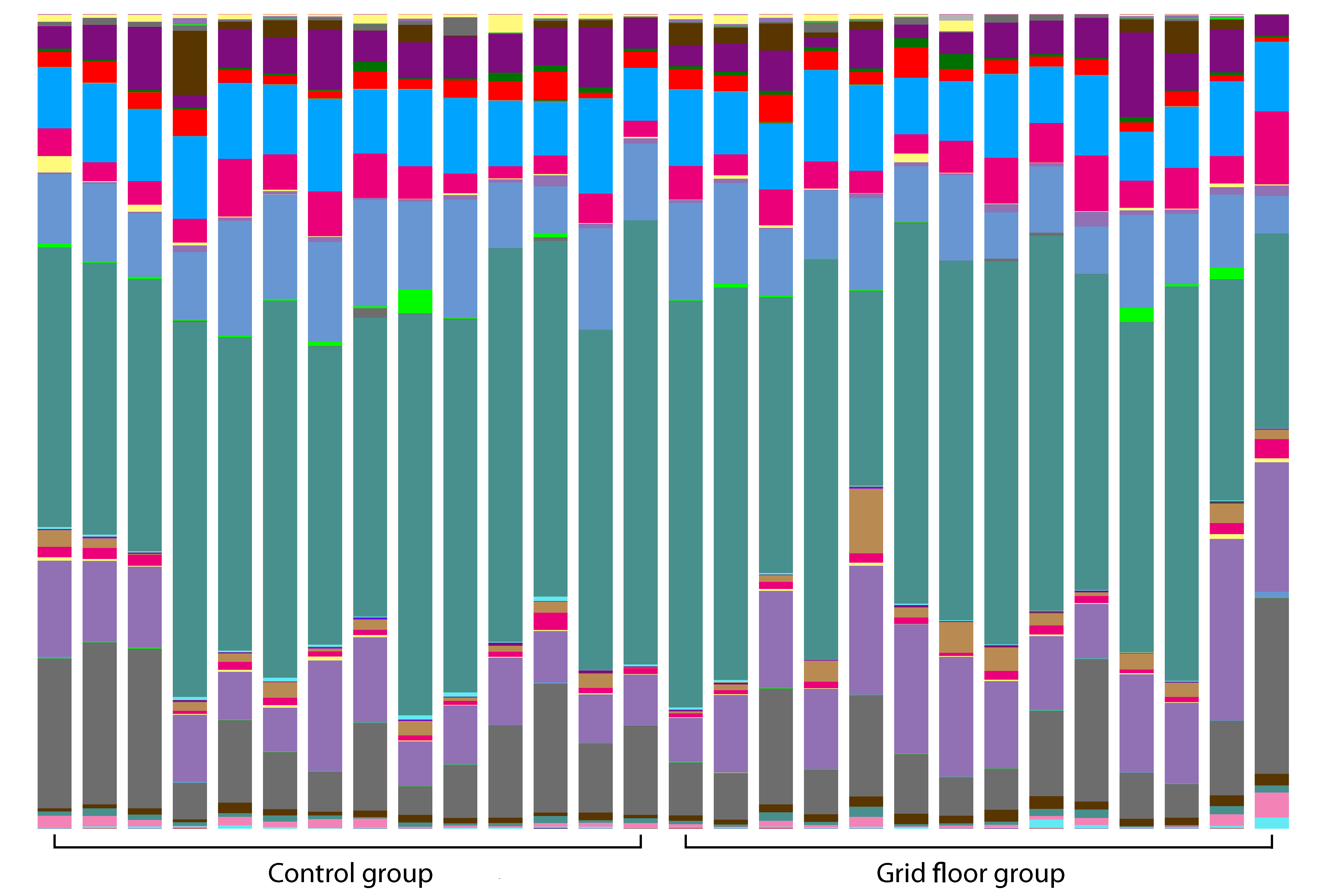

Supplement: Figure S2 — Relative distribution of genera. Bar chart presenting the relative distribution (% of total) of all 77 genera found in the cecum within the two groups of mice as determined by 16S rRNA gene 454/FLX based pyrosequencing. See Table S1 for colour legends. (TIF) [file pone.0046231.s002.tif]

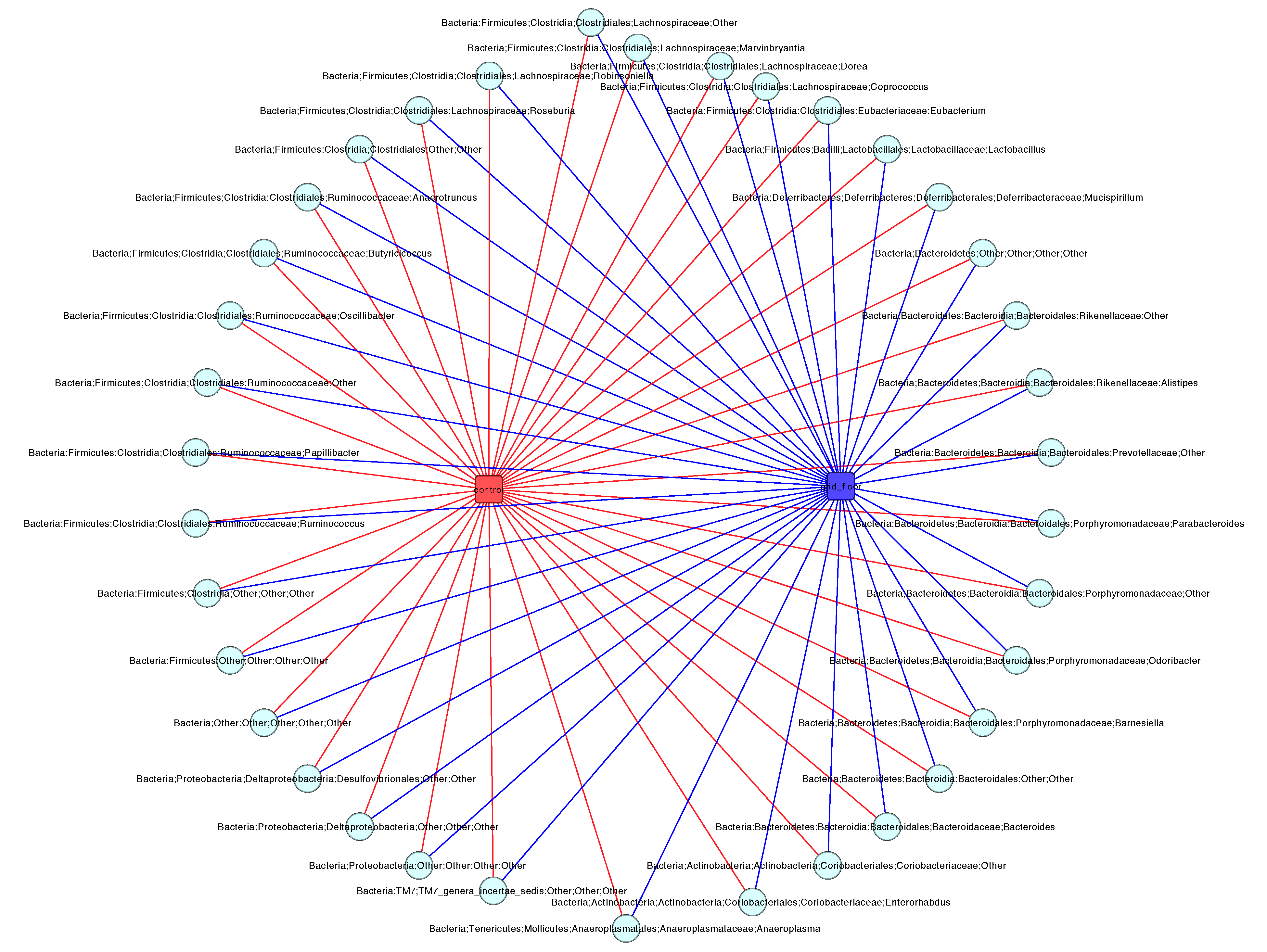

Supplement: Figure S3 — Shared genera. Shared genera between the control group (red) and grid floor housed mice (blue) as determined by 16S rRNA gene 454/FLX based pyrosequencing. The circle nodes denote the 36 genera that reached the abundance threshold of 0.003% within each group. As seen all genera present above the threshold value are represented in both groups of mice. (TIF) [file pone.0046231.s003.tif]
