# Supplementary material for: Gut Microbiota Composition Is Correlated to Grid Floor Induced Stress and Behavior in the BALB/c Mouse
Source: PLoS One. 2012 Oct 2;7(10):e46231. doi: 10.1371/journal.pone.0046231 (PMC3462757; doi:10.1371/journal.pone.0046231)
Supplement: Table S1 — Relative distribution (% of total) cecum microbiota as determined by 16S rRNA gene 454/FLX based pyrosequencing for each mouse. The last four columns include the mean values for each genus in the groups and the significantly different taxa between categories analysed with Metastats and the Wilcoxon rank sum test performed for 1000 subsampled OTU tables. Colour codes refer to Figure S1. (PDF) [file pone.0046231.s004.pdf]

Legend	Taxonomy	1<br>control1<br>%	2<br>control2<br>%	3<br>control3<br>%	4<br>control4<br>%	5<br>control5<br>%	6<br>control6<br>%	7<br>control7<br>%	8<br>control8<br>%	9<br>control9<br>%	10<br>control10<br>%	11<br>control11<br>%	12<br>control12<br>%	13<br>control13<br>%	14<br>control14<br>%	15<br>grid floor1<br>%	16<br>grid floor2<br>%	17<br>grid floor3<br>%	18<br>grid floor4<br>%	19<br>grid floor5<br>%	20<br>grid floor6<br>%	21<br>grid floor7<br>%	22<br>grid floor8<br>%	23<br>grid floor9<br>%	24<br>grid floor10<br>%	25<br>grid floor11<br>%	26<br>grid floor12<br>%	27<br>grid floor13<br>%	28<br>grid floor14<br>%	control<br>mean	grid floor<br>mean	significantly<br>different
	Bacteria;Actinobacteria;Actinobacteria;Actinomycetales;Other;Other	0.0000000000	0.0000000000	0.0000000000	0.0000000000	0.0000000000	0.0000000000	0.0000000000	0.0000000000	0.0000000000	0.0000000000	0.0000000000	0.0000000000	0.0000000000	0.0000000000	0.0000000000	0.0000000000	0.0000000000	0.0000000000	0.0001065303	0.0000000000	0.0000000000	0.0000000000	0.0000000000	0.0000000000	0.0000000000	0.0000000000	0.0000000000	0.0000000000	0.0000000000	0.0000076093	no
	Bacteria;Actinobacteria;Actinobacteria;Bifidobacteriales;Bifidobacteriaceae;Bifidobacterium	0.0000000000	0.0000000000	0.0000000000	0.0000000000	0.0000000000	0.0000000000	0.0000000000	0.0000000000	0.0000000000	0.0000000000	0.0000000000	0.0002491591	0.0001266464	0.0000000000	0.0000000000	0.0000000000	0.0002152158	0.0000000000	0.0000000000	0.0000000000	0.0000000000	0.0000000000	0.0000000000	0.0000000000	0.0000000000	0.0000000000	0.0000000000	0.0000000000	0.0000268432	0.0000153726	no
	Bacteria;Actinobacteria;Actinobacteria;Coriobacteriales;Coriobacteriaceae;Enterorhabdus	0.0002184837	0.0003949967	0.0001626545	0.0004081077	0.0000000000	0.0000000000	0.0000000000	0.0000000000	0.0000000000	0.0000000000	0.0000000000	0.0002491591	0.0000000000	0.0003475440	0.0002585093	0.0000946342	0.0002152158	0.0001079331	0.0001065303	0.0000000000	0.0001143772	0.0001100715	0.0002960332	0.0000000000	0.0000000000	0.0001104240	0.0003808073	0.0000000000	0.0001272104	0.0001281811	no
	Bacteria;Actinobacteria;Actinobacteria;Coriobacteriales;Coriobacteriaceae;Olsenella	0.0000000000	0.0000000000	0.0000000000	0.0000000000	0.0000000000	0.0000000000	0.0000000000	0.0000000000	0.0000000000	0.0000000000	0.0000000000	0.0002491591	0.0000000000	0.0000000000	0.0000000000	0.0000000000	0.0000000000	0.0000000000	0.0000000000	0.0000000000	0.0000000000	0.0000000000	0.0000000000	0.0000000000	0.0000000000	0.0000000000	0.0000000000	0.0000000000	0.0000177971	0.0000000000	no
	Bacteria;Actinobacteria;Actinobacteria;Coriobacteriales;Coriobacteriaceae;Other	0.0002184837	0.0003949967	0.0001626545	0.0005441437	0.0000000000	0.0000000000	0.0000000000	0.0002566076	0.0002458513	0.0001382170	0.0001306506	0.0004983182	0.0001266464	0.0004633920	0.0006893580	0.0003785370	0.0005380394	0.0004317323	0.0003195909	0.0001251564	0.0004575089	0.0007705008	0.0005920663	0.0005173841	0.0002886419	0.0004416961	0.0003808073	0.0000000000	0.0002271401	0.0004236443	yes
	Bacteria;Actinobacteria;Actinobacteria;Other;Other;Other	0.0000000000	0.0000000000	0.0000000000	0.0000000000	0.0000000000	0.0000000000	0.0000000000	0.0001283038	0.0000000000	0.0000000000	0.0000000000	0.0000000000	0.0000000000	0.0000000000	0.0000000000	0.0000000000	0.0000000000	0.0000000000	0.0000000000	0.0000000000	0.0000000000	0.0000000000	0.0000000000	0.0000000000	0.0000000000	0.0000000000	0.0000000000	0.0000000000	0.0000091646	0.0000000000	no
	Bacteria;Bacteroidetes;Bacteroidia;Bacteroidales;Bacteroidaceae;Bacteroides	0.0006554512	0.0019749835	0.0024398178	0.0020405387	0.0043495990	0.0020102057	0.0007859164	0.0011547344	0.0003687769	0.0009675190	0.0013065064	0.0013703750	0.0021529889	0.0008109361	0.0010340371	0.0010409766	0.0016141182	0.0012951970	0.0019175455	0.0018773467	0.0003431316	0.0013208586	0.0106571936	0.0039321192	0.0008659258	0.0005521201	0.0026656512	0.0138625592	0.0015991678	0.0030699129	no
	Bacteria;Bacteroidetes;Bacteroidia;Bacteroidales;Other;Other	0.0148568932	0.0129032258	0.0081327261	0.0004081077	0.0103302977	0.0068037730	0.0111600126	0.0109058250	0.0019668101	0.0027643400	0.0018291090	0.0042357045	0.0049392097	0.0054448563	0.0037914692	0.0011356109	0.0073173356	0.0028062601	0.0123575157	0.0008760951	0.0028594304	0.0028618602	0.0040457865	0.0087955298	0.0008659258	0.0017667845	0.0142168063	0.0308056872	0.0069057779	0.0067501498	no
	Bacteria;Bacteroidetes;Bacteroidia;Bacteroidales;Porphyromonadaceae;Barnesiella	0.0054620931	0.0093482554	0.0066688354	0.0044891851	0.0044855240	0.0077315602	0.0045583150	0.0017962535	0.0050399508	0.0026261230	0.0039195192	0.0087205681	0.0032928065	0.0057924004	0.0038776389	0.0024604902	0.0103303562	0.0036697248	0.0123575157	0.0027534418	0.0027450532	0.0038525041	0.0087823170	0.0103476821	0.0011545678	0.0015459364	0.0102817974	0.0084123223	0.0052808136	0.0058979534	no
	Bacteria;Bacteroidetes;Bacteroidia;Bacteroidales;Porphyromonadaceae;Odoribacter	0.0036049814	0.0050032916	0.0074821080	0.0036729697	0.0129128721	0.0075769290	0.0044011317	0.0079548371	0.0093423479	0.0066344160	0.0064018814	0.0041111250	0.0093718338	0.0041705283	0.0066350711	0.0059619570	0.0097923168	0.0096060443	0.0123575157	0.0128911139	0.0094933089	0.0143093010	0.0155910795	0.0098302980	0.0090922211	0.0090547703	0.0130743844	0.0144549763	0.0066172324	0.0108674542	yes
	Bacteria;Bacteroidetes;Bacteroidia;Bacteroidales;Porphyromonadaceae;Other	0.1841817785	0.1988150099	0.1963240078	0.0447558155	0.1014000272	0.0702025669	0.0493555486	0.1079035155	0.0350338045	0.0653766413	0.1135354063	0.1585897596	0.0848530902	0.1097080630	0.0651443343	0.0571590802	0.1423652211	0.0547220723	0.1245339299	0.0732165207	0.0472377902	0.0503026968	0.1048944148	0.1748758278	0.0567181411	0.0412985866	0.0916476263	0.2158767773	0.1085739311	0.0928566442	no
	Bacteria;Bacteroidetes;Bacteroidia;Bacteroidales;Porphyromonadaceae;Parabacteroides	0.0003277256	0.0003949967	0.0008132726	0.0000000000	0.0002718499	0.0003092624	0.0001571833	0.0001283038	0.0004917025	0.0002764340	0.0000000000	0.0001245795	0.0003799392	0.0004633920	0.0001723395	0.0001892685	0.0005380394	0.0005396654	0.0001065303	0.0003754693	0.0002287544	0.0004402862	0.0002960332	0.0006208609	0.0000000000	0.0000000000	0.0001269358	0.0009478673	0.0002956173	0.0003272893	no
	Bacteria;Bacteroidetes;Bacteroidia;Bacteroidales;Prevotellaceae;Hallella	0.0000000000	0.0000000000	0.0000000000	0.0000000000	0.0000000000	0.0000000000	0.0000000000	0.0000000000	0.0000000000	0.0000000000	0.0000000000	0.0000000000	0.0000000000	0.0000000000	0.0000000000	0.0000000000	0.0000000000	0.0000000000	0.0000000000	0.0000000000	0.0000000000	0.0000000000	0.0000000000	0.0000000000	0.0000000000	0.0000000000	0.0000000000	0.0001184834	0.0000000000	0.0000084631	no
	Bacteria;Bacteroidetes;Bacteroidia;Bacteroidales;Prevotellaceae;Other	0.0015293861	0.0007899934	0.0004879636	0.0010882873	0.0006796248	0.0004638936	0.0009430997	0.0003849115	0.0001229256	0.0005528680	0.0005226026	0.0013703750	0.0000000000	0.0003475440	0.0011202068	0.0005678054	0.0005380394	0.0003237992	0.0005326515	0.0006257822	0.0003431316	0.0002201431	0.0009867772	0.0010347682	0.0005772839	0.0011042403	0.0007616146	0.0066350711	0.0006631054	0.0010979510	no
	Bacteria;Bacteroidetes;Bacteroidia;Bacteroidales;Prevotellaceae;Paraprevotella	0.0000000000	0.0000000000	0.0000000000	0.0000000000	0.0000000000	0.0000000000	0.0000000000	0.0000000000	0.0000000000	0.0000000000	0.0000000000	0.0001245795	0.0000000000	0.0000000000	0.0000000000	0.0000946342	0.0000000000	0.0000000000	0.0000000000	0.0000000000	0.0000000000	0.0000000000	0.0000000000	0.0000000000	0.0000000000	0.0000000000	0.0000000000	0.0000000000	0.0000088985	0.0000067596	no
	Bacteria;Bacteroidetes;Bacteroidia;Bacteroidales;Prevotellaceae;Prevotella	0.0000000000	0.0000000000	0.0000000000	0.0002720718	0.0000000000	0.0000000000	0.0000000000	0.0000000000	0.0000000000	0.0000000000	0.0000000000	0.0000000000	0.0000000000	0.0001158480	0.0000000000	0.0000000000	0.0000000000	0.0000000000	0.0000000000	0.0000000000	0.0000000000	0.0000000000	0.0000000000	0.0000000000	0.0000000000	0.0000000000	0.0000000000	0.0000000000	0.0000277086	0.0000000000	no
	Bacteria;Bacteroidetes;Bacteroidia;Bacteroidales;Rikenellaceae;Alistipes	0.1180904523	0.0987491771	0.0992192583	0.0823017277	0.0583118119	0.0536570280	0.1351776171	0.1045676161	0.0545789797	0.0720110574	0.0824405540	0.0630372493	0.0599037487	0.0615152919	0.0533390780	0.0953913126	0.1186914882	0.0983270372	0.1583040375	0.1583229036	0.1474322315	0.1072096863	0.0904874679	0.0662251656	0.1200750469	0.0987190813	0.2223914699	0.1590047393	0.0816829692	0.1209943390	yes
	Bacteria;Bacteroidetes;Bacteroidia;Bacteroidales;Rikenellaceae;Other	0.0041511907	0.0026333114	0.0009759271	0.0010882873	0.0024466494	0.0030926241	0.0050298648	0.0026943803	0.0012292563	0.0008293020	0.0011758558	0.0011212159	0.0013931104	0.0009267841	0.0008616975	0.0008517081	0.0024749812	0.0008634647	0.0039416214	0.0003754693	0.0010293949	0.0016510732	0.0018748767	0.0011382450	0.0012988887	0.0007729682	0.0058390454	0.0048578199	0.0020562685	0.0019879467	no
	Bacteria;Bacteroidetes;Flavobacteria;Flavobacteriales;Flavobacteriaceae;Chryseobacterium	0.0000000000	0.0000000000	0.0000000000	0.0000000000	0.0000000000	0.0000000000	0.0000000000	0.0000000000	0.0000000000	0.0000000000	0.0000000000	0.0000000000	0.0000000000	0.0000000000	0.0000000000	0.0000000000	0.0000000000	0.0000000000	0.0000000000	0.0000000000	0.0000000000	0.0000000000	0.0000000000	0.0000000000	0.0000000000	0.0001104240	0.0000000000	0.0000000000	0.0000000000	0.0000078874	no
	Bacteria;Bacteroidetes;Other;Other;Other;Other	0.0128905397	0.0132982225	0.0136629798	0.0039450415	0.0096506728	0.0088139787	0.0062873310	0.0062868873	0.0062692071	0.0049758120	0.0061405801	0.0211785225	0.0063323202	0.0072984245	0.0046531667	0.0047317119	0.0085010223	0.0080949811	0.0112922126	0.0080100125	0.0037744481	0.0108970831	0.0113479376	0.0085885762	0.0044739501	0.0065150177	0.0137090632	0.0234597156	0.0090736086	0.0091463499	no
	Bacteria;Cyanobacteria;Cyanobacteria;Chloroplast;Streptophyta;Other	0.0000000000	0.0000000000	0.0000000000	0.0000000000	0.0000000000	0.0000000000	0.0000000000	0.0003849115	0.0000000000	0.0000000000	0.0000000000	0.0000000000	0.0000000000	0.0000000000	0.0000000000	0.0000000000	0.0000000000	0.0000000000	0.0000000000	0.0000000000	0.0001143772	0.0000000000	0.0000000000	0.0000000000	0.0000000000	0.0000000000	0.0000000000	0.0000000000	0.0000274937	0.0000081698	no
	Bacteria;Deferribacteres;Deferribacteres;Deferribacterales;Deferribacteraceae;Mucispirillum	0.0204282281	0.0121132324	0.0011385817	0.0106108013	0.0106021476	0.0191742694	0.0034580321	0.0124454709	0.0177012907	0.0041465100	0.0071857852	0.0137037498	0.0179837893	0.0006950880	0.0027574321	0.0070975679	0.0082858065	0.0255801403	0.0795781400	0.0122653317	0.0376301041	0.0288387452	0.0147029801	0.0043460265	0.0197719729	0.0175574205	0.0238639249	0.0113744076	0.0108133555	0.0209750000	no
	Bacteria;Firmicutes;Bacilli;Bacillales;Staphylococcaceae;Gemella	0.0000000000	0.0000000000	0.0000000000	0.0000000000	0.0000000000	0.0000000000	0.0000000000	0.0000000000	0.0001229256	0.0000000000	0.0000000000	0.0000000000	0.0000000000	0.0000000000	0.0000000000	0.0000000000	0.0000000000	0.0000000000	0.0000000000	0.0000000000	0.0000000000	0.0000000000	0.0000986777	0.0000000000	0.0000000000	0.0000000000	0.0000000000	0.0000000000	0.0000087804	0.0000070484	no
	Bacteria;Firmicutes;Bacilli;Lactobacillales;Lactobacillaceae;Lactobacillus	0.0005462093	0.0005266623	0.0008132726	0.0004081077	0.0001359250	0.0004638936	0.0007859164	0.0012830382	0.0004917025	0.0002764340	0.0001306506	0.0003737386	0.0001266464	0.0006950880	0.0009478673	0.0002839027	0.0002152158	0.0004317323	0.0014914243	0.0000000000	0.0000000000	0.0001100715	0.0002960332	0.0000000000	0.0001443210	0.0000000000	0.0008885504	0.0000000000	0.0005040918	0.0003435085	no
	Bacteria;Firmicutes;Bacilli;Lactobacillales;Leuconostocaceae;Weissella	0.0000000000	0.0000000000	0.0000000000	0.0000000000	0.0000000000	0.0000000000	0.0000000000	0.0000000000	0.0000000000	0.0000000000	0.0000000000	0.0000000000	0.0000000000	0.0000000000	0.0000000000	0.0000000000	0.0000000000	0.0001079331	0.0000000000	0.0000000000	0.0000000000	0.0000000000	0.0000000000	0.0000000000	0.0000000000	0.0000000000	0.0000000000	0.0002369668	0.0000000000	0.0000246357	no
	Bacteria;Firmicutes;Bacilli;Lactobacillales;Streptococcaceae;Lactococcus	0.0000000000	0.0000000000	0.0000000000	0.0001360359	0.0000000000	0.0000000000	0.0000000000	0.0001283038	0.0000000000	0.0001382170	0.0000000000	0.0000000000	0.0000000000	0.0000000000	0.0000000000	0.0000946342	0.0000000000	0.0000000000	0.0000000000	0.0000000000	0.0000000000	0.0000000000	0.0002960332	0.0000000000	0.0000000000	0.0000000000	0.0001269358	0.0000000000	0.0000287541	0.0000369717	no
	Bacteria;Firmicutes;Bacilli;Lactobacillales;Streptococcaceae;Other	0.0000000000	0.0000000000	0.0000000000	0.0000000000	0.0000000000	0.0000000000	0.0000000000	0.0000000000	0.0000000000	0.0000000000	0.0000000000	0.0000000000	0.0000000000	0.0000000000	0.0000861698	0.0000000000	0.0000000000	0.0000000000	0.0000000000	0.0000000000	0.0000000000	0.0000000000	0.0000000000	0.0000000000	0.0000000000	0.0000000000	0.0000000000	0.0000000000	0.0000000000	0.0000061550	no
	Bacteria;Firmicutes;Bacilli;Lactobacillales;Streptococcaceae;Streptococcus	0.0000000000	0.0000000000	0.0000000000	0.0000000000	0.0001359250	0.0000000000	0.0000000000	0.0001283038	0.0000000000	0.0000000000	0.0001306506	0.0000000000	0.0000000000	0.0000000000	0.0000000000	0.0000000000	0.0001076079	0.0000000000	0.0000000000	0.0000000000	0.0000000000	0.0000000000	0.0000000000	0.0001034768	0.0000000000	0.0000000000	0.0000000000	0.0001184834	0.0000282057	0.0000235406	no
	Bacteria;Firmicutes;Clostridia;Clostridiales;Eubacteriaceae;Eubacterium	0.0002184837	0.0005266623	0.0004879636	0.0001360359	0.0000000000	0.0000000000	0.0000000000	0.0000000000	0.0000000000	0.0001382170	0.0002613013	0.0001245795	0.0003799392	0.0002316960	0.0000861698	0.0002839027	0.0000000000	0.0001079331	0.0005326515	0.0000000000	0.0000000000	0.0003302146	0.0003947109	0.0001034768	0.0000000000	0.0001104240	0.0005077431	0.0001184834	0.0001789199	0.0001839793	no
	Bacteria;Firmicutes;Clostridia;Clostridiales;Incertae Sedis XIV;Blautia	0.0000000000	0.0001316656	0.0000000000	0.0000000000	0.0000000000	0.0000000000	0.0000000000	0.0000000000	0.0000000000	0.0000000000	0.0000000000	0.0000000000	0.0000000000	0.0000000000	0.0000000000	0.0000000000	0.0000000000	0.0000000000	0.0000000000	0.0000000000	0.0000000000	0.0000000000	0.0000000000	0.0000000000	0.0000000000	0.0000000000	0.0000000000	0.0000000000	0.0000094047	0.0000000000	no
	Bacteria;Firmicutes;Clostridia;Clostridiales;Lachnospiraceae;Acetitomaculum	0.0001092419	0.0000000000	0.0000000000	0.0000000000	0.0000000000	0.0000000000	0.0000000000	0.0001283038	0.0000000000	0.0000000000	0.0000000000	0.0000000000	0.0000000000	0.0000000000	0.0000000000	0.0000000000	0.0000000000	0.0000000000	0.0000000000	0.0000000000	0.0000000000	0.0000000000	0.0000000000	0.0000000000	0.0000000000	0.0001104240	0.0000000000	0.0001184834	0.0000169675	0.0000163505	no
	Bacteria;Firmicutes;Clostridia;Clostridiales;Lachnospiraceae;Anaerosporobacter	0.0000000000	0.0000000000	0.0000000000	0.0000000000	0.0000000000	0.0003092624	0.0001571833	0.0001283038	0.0000000000	0.0000000000	0.0000000000	0.0000000000	0.0000000000	0.0000000000	0.0000861698	0.0000946342	0.0000000000	0.0000000000	0.0000000000	0.0001251564	0.0001143772	0.0001100715	0.0000000000	0.0000000000	0.0000000000	0.0000000000	0.0000000000	0.0001184834	0.0000424821	0.0000463495	no
	Bacteria;Firmicutes;Clostridia;Clostridiales;Lachnospiraceae;Coprococcus	0.0001092419	0.0000000000	0.0000000000	0.0000000000	0.0000000000	0.0001546312	0.0000000000	0.0001283038	0.0000000000	0.0000000000	0.0000000000	0.0001245795	0.0001266464	0.0000000000	0.0000861698	0.0000000000	0.0000000000	0.0001079331	0.0000000000	0.0001251564	0.0000000000	0.0001100715	0.0000000000	0.0000000000	0.0000000000	0.0000000000	0.0001269358	0.0000000000	0.0000459573	0.0000397333	no
	Bacteria;Firmicutes;Clostridia;Clostridiales;Lachnospiraceae;Dorea	0.0005462093	0.0007899934	0.0001626545	0.0016324310	0.0009514748	0.0003092624	0.0009430997	0.0005132153	0.0009834050	0.0004146510	0.0032662660	0.0001245795	0.0029128673	0.0004633920	0.0006893580	0.0016087821	0.0003228236	0.0006475985	0.0005326515	0.0026282854	0.0010293949	0.0020913594	0.0013814881	0.0019660596	0.0008659258	0.0007729682	0.0012693577	0.0007109005	0.0010009644	0.0011797824	no
	Bacteria;Firmicutes;Clostridia;Clostridiales;Lachnospiraceae;Johnsonella	0.0000000000	0.0000000000	0.0000000000	0.0000000000	0.0000000000	0.0000000000	0.0000000000	0.0001283038	0.0000000000	0.0000000000	0.0000000000	0.0000000000	0.0000000000	0.0000000000	0.0000861698	0.0002839027	0.0000000000	0.0000000000	0.0000000000	0.0000000000	0.0000000000	0.0000000000	0.0000000000	0.0000000000	0.0001443210	0.0000000000	0.0000000000	0.0000000000	0.0000091646	0.0000367424	no
	Bacteria;Firmicutes;Clostridia;Clostridiales;Lachnospiraceae;Marvinbryantia	0.0022940791	0.0023699803	0.0014638907	0.0039450415	0.0023107245	0.0044843049	0.0028292990	0.0008981268	0.0054087277	0.0052522460	0.0011758558	0.0054814999	0.0006332320	0.0022011121	0.0025850926	0.0027443929	0.0020445497	0.0002158662	0.0007457122	0.0020025031	0.0010293949	0.0009906439	0.0004933886	0.0006208609	0.0002886419	0.0009938163	0.0007616146	0.0004739336	0.0029105800	0.0011421723	yes
	Bacteria;Firmicutes;Clostridia;Clostridiales;Lachnospiraceae;Moryella	0.0000000000	0.0000000000	0.0000000000	0.0000000000	0.0000000000	0.0000000000	0.0000000000	0.0000000000	0.0000000000	0.0000000000	0.0000000000	0.0000000000	0.0000000000	0.0000000000	0.0000000000	0.0000000000	0.0000000000	0.0000000000	0.0000000000	0.0000000000	0.0000000000	0.0000000000	0.0001973554	0.0000000000	0.0000000000	0.0000000000	0.0000000000	0.0001184834	0.0000000000	0.0000225599	no
	Bacteria;Firmicutes;Clostridia;Clostridiales;Lachnospiraceae;Other	0.3434564125	0.3333772219	0.3340923878	0.4603455312	0.3849395134	0.4631204577	0.3659226658	0.3670772389	0.4923171481	0.4580511403	0.4835380193	0.4369004609	0.4178064843	0.5455282669	0.4995260664	0.4819721775	0.3384267728	0.4914193200	0.2394801321	0.4668335419	0.4413816768	0.4704457898	0.4603315571	0.3879346026	0.4052532833	0.4846510601	0.2708809342	0.2388625592	0.4204623535	0.4055285339	no
	Bacteria;Firmicutes;Clostridia;Clostridiales;Lachnospiraceae;Pseudobutyrivibrio	0.0000000000	0.0000000000	0.0001626545	0.0000000000	0.0000000000	0.0000000000	0.0000000000	0.0000000000	0.0000000000	0.0000000000	0.0000000000	0.0000000000	0.0000000000	0.0000000000	0.0000000000	0.0000000000	0.0000000000	0.0000000000	0.0000000000	0.0000000000	0.0000000000	0.0000000000	0.0000000000	0.0000000000	0.0000000000	0.0000000000	0.0000000000	0.0000000000	0.0000116182	0.0000000000	no
	Bacteria;Firmicutes;Clostridia;Clostridiales;Lachnospiraceae;Robinsoniella	0.0001092419	0.0002633311	0.0001626545	0.0016324310	0.0001359250	0.0001546312	0.0009430997	0.0119322556	0.0011063307	0.0009675190	0.0000000000	0.0042357045	0.0001266464	0.0000000000	0.0002585093	0.0002839027	0.0004304315	0.0000000000	0.0001065303	0.0011264080	0.0001143772	0.0030820033	0.0038484310	0.0002069536	0.0002886419	0.0000000000	0.0007616146	0.0002369668	0.0015549836	0.0007674836	no
	Bacteria;Firmicutes;Clostridia;Clostridiales;Lachnospiraceae;Roseburia	0.0048066419	0.0010533246	0.0029277814	0.0016324310	0.0014951747	0.0018555745	0.0055014146	0.0021811650	0.0293792256	0.0012439530	0.0002613013	0.0053569204	0.0000000000	0.0000000000	0.0012925463	0.0043531750	0.0015065103	0.0004317323	0.0018110152	0.0007509387	0.0000000000	0.0000000000	0.0001973554	0.0000000000	0.0176071583	0.0026501767	0.0147245494	0.0003554502	0.0041210648	0.0032629006	no
	Bacteria;Firmicutes;Clostridia;Clostridiales;Lachnospiraceae;Syntrophococcus	0.0000000000	0.0000000000	0.0000000000	0.0000000000	0.0001359250	0.0000000000	0.0000000000	0.0001283038	0.0000000000	0.0000000000	0.0000000000	0.0000000000	0.0000000000	0.0000000000	0.0000000000	0.0000000000	0.0000000000	0.0000000000	0.0000000000	0.0000000000	0.0000000000	0.0000000000	0.0000000000	0.0000000000	0.0000000000	0.0000000000	0.0000000000	0.0000000000	0.0000188735	0.0000000000	no
	Bacteria;Firmicutes;Clostridia;Clostridiales;Other;Other	0.0850994101	0.0961158657	0.0779115159	0.0825737995	0.1402745684	0.1283438998	0.1222885885	0.1303566846	0.1079287031	0.1447131997	0.0802194931	0.0571820107	0.1244934144	0.0943002780	0.1186557518	0.1238762184	0.0818895943	0.0841878036	0.1119633536	0.0682102628	0.1053414160	0.0567969180	0.0810144069	0.0583609272	0.1140135662	0.0863515901	0.0893627824	0.0459715640	0.1051286737	0.0875711540	no
	Bacteria;Firmicutes;Clostridia;Clostridiales;Ruminococcaceae;Acetanaerobacterium	0.0000000000	0.0000000000	0.0000000000	0.0000000000	0.0000000000	0.0001546312	0.0000000000	0.0000000000	0.0000000000	0.0000000000	0.0000000000	0.0000000000	0.0000000000	0.0000000000	0.0000000000	0.0000000000	0.0000000000	0.0000000000	0.0000000000	0.0000000000	0.0000000000	0.0000000000	0.0000000000	0.0000000000	0.0000000000	0.0000000000	0.0000000000	0.0000000000	0.0000110451	0.0000000000	no
	Bacteria;Firmicutes;Clostridia;Clostridiales;Ruminococcaceae;Acetivibrio	0.0000000000	0.0000000000	0.0001626545	0.0000000000	0.0001359250	0.0000000000	0.0000000000	0.0000000000	0.0000000000	0.0001382170	0.0000000000	0.0000000000	0.0000000000	0.0000000000	0.0000000000	0.0000000000	0.0000000000	0.0000000000	0.0000000000	0.0000000000	0.0000000000	0.0000000000	0.0000000000	0.0000000000	0.0000000000	0.0000000000	0.0000000000	0.0000000000	0.0000311997	0.0000000000	no
	Bacteria;Firmicutes;Clostridia;Clostridiales;Ruminococcaceae;Anaerotruncus	0.0019663535	0.0022383147	0.0016265452	0.0084342266	0.0046214490	0.0040204113	0.0061301478	0.0026943803	0.0031960664	0.0056668970	0.0039195192	0.0135791703	0.0055724417	0.0064874884	0.0034467902	0.0053941516	0.0016141182	0.0008634647	0.0059656972	0.0050062578	0.0021731671	0.0102366538	0.0047365305	0.0181084437	0.0054841969	0.0054107774	0.0090124397	0.0126777251	0.0050109580	0.0064378867	no
	Bacteria;Firmicutes;Clostridia;Clostridiales;Ruminococcaceae;Butyricicoccus	0.0202097444	0.0010533246	0.0086206897	0.0034008978	0.0009514748	0.0015463120	0.0007859164	0.0000000000	0.0004917025	0.0020732550	0.0013065064	0.0014949545	0.0007598784	0.0018535681	0.0005170185	0.0039746380	0.0032282363	0.0014031301	0.0002130606	0.0107634543	0.0006862633	0.0006604293	0.0001973554	0.0006208609	0.0031750613	0.0009938163	0.0046966235	0.0014218009	0.0031820160	0.0023251249	no
	Bacteria;Firmicutes;Clostridia;Clostridiales;Ruminococcaceae;Hydrogenoanaerobacterium	0.0001092419	0.0000000000	0.0000000000	0.0000000000	0.0000000000	0.0000000000	0.0001571833	0.0000000000	0.0000000000	0.0002764340	0.0000000000	0.0002491591	0.0000000000	0.0000000000	0.0000000000	0.0000946342	0.0000000000	0.0000000000	0.0000000000	0.0000000000	0.0000000000	0.0000000000	0.0000986777	0.0000000000	0.0000000000	0.0000000000	0.0000000000	0.0001184834	0.0000565727	0.0000222711	no
	Bacteria;Firmicutes;Clostridia;Clostridiales;Ruminococcaceae;Oscillibacter	0.0339742189	0.0231731402	0.0291151594	0.0291116855	0.0709528340	0.0432967373	0.0548569632	0.0542725173	0.0398279041	0.0240497581	0.0147635223	0.0227980566	0.0360942249	0.0195783133	0.0411891426	0.0256458787	0.0437964059	0.0332433891	0.0273782891	0.0236545682	0.0391170079	0.0561364887	0.0484507598	0.0685016556	0.0333381440	0.0498012367	0.0337649150	0.0893364929	0.0354189311	0.0438110267	no
	Bacteria;Firmicutes;Clostridia;Clostridiales;Ruminococcaceae;Other	0.0749399170	0.0965108624	0.0876707872	0.1014827915	0.0922930542	0.0856656873	0.1134863251	0.0783936361	0.0937922557	0.0927436075	0.0802194931	0.0652796811	0.1168946302	0.0649907322	0.0933218440	0.0765590991	0.0809211234	0.1120345386	0.1049323533	0.0692115144	0.0728582866	0.1026967529	0.0691730807	0.0985099338	0.0597488815	0.0750883392	0.0917745621	0.0850710900	0.0888831043	0.0851358143	no
	Bacteria;Firmicutes;Clostridia;Clostridiales;Ruminococcaceae;Papillibacter	0.0003277256	0.0013166557	0.0003253090	0.0001360359	0.0006796248	0.0004638936	0.0006287331	0.0012830382	0.0008604794	0.0001382170	0.0011758558	0.0003737386	0.0003799392	0.0001158480	0.0008616975	0.0007570739	0.0001076079	0.0003237992	0.0006391818	0.0000000000	0.0005718861	0.0005503577	0.0004933886	0.0004139073	0.0004329629	0.0009938163	0.0002538715	0.0005924171	0.0005860781	0.0004994263	no
	Bacteria;Firmicutes;Clostridia;Clostridiales;Ruminococcaceae;Ruminococcus	0.0003277256	0.0001316656	0.0009759271	0.0001360359	0.0005436999	0.0001546312	0.0006287331	0.0000000000	0.0006146281	0.0005528680	0.0000000000	0.0017441136	0.0000000000	0.0000000000	0.0003446790	0.0000000000	0.0022597654	0.0000000000	0.0011718334	0.0000000000	0.0000000000	0.0001100715	0.0000000000	0.0000000000	0.0001443210	0.0000000000	0.0000000000	0.0002369668	0.0004150020	0.0003048312	no
	Bacteria;Firmicutes;Clostridia;Clostridiales;Ruminococcaceae;Sporobacter	0.0000000000	0.0001316656	0.0000000000	0.0000000000	0.0001359250	0.0000000000	0.0000000000	0.0000000000	0.0000000000	0.0001382170	0.0001306506	0.0000000000	0.0001266464	0.0000000000	0.0000000000	0.0000000000	0.0000000000	0.0000000000	0.0001065303	0.0000000000	0.0001143772	0.0000000000	0.0000000000	0.0000000000	0.0000000000	0.0000000000	0.0001269358	0.0000000000	0.0000473646	0.0000248459	no
	Bacteria;Firmicutes;Clostridia;Clostridiales;Veillonellaceae;Megasphaera	0.0000000000	0.0000000000	0.0000000000	0.0000000000	0.0000000000	0.0000000000	0.0000000000	0.0000000000	0.0000000000	0.0000000000	0.0000000000	0.0001245795	0.0000000000	0.0000000000	0.0000000000	0.0000000000	0.0000000000	0.0000000000	0.0000000000	0.0000000000	0.0000000000	0.0000000000	0.0000000000	0.0000000000	0.0000000000	0.0000000000	0.0000000000	0.0000000000	0.0000088985	0.0000000000	no
	Bacteria;Firmicutes;Clostridia;Clostridiales;Veillonellaceae;Other	0.0000000000	0.0000000000	0.0000000000	0.0000000000	0.0000000000	0.0000000000	0.0000000000	0.0000000000	0.0000000000	0.0000000000	0.0000000000	0.0004983182	0.0000000000	0.0001158480	0.0000000000	0.0001892685	0.0000000000	0.0000000000	0.0000000000	0.0000000000	0.0000000000	0.0000000000	0.0000000000	0.0000000000	0.0000000000	0.0000000000	0.0000000000	0.0000000000	0.0000438690	0.0000135192	no
	Bacteria;Firmicutes;Clostridia;Clostridiales;Veillonellaceae;Phascolarctobacterium	0.0000000000	0.0000000000	0.0000000000	0.0000000000	0.0000000000	0.0000000000	0.0000000000	0.0000000000	0.0000000000	0.0000000000	0.0000000000	0.0000000000	0.0000000000	0.0000000000	0.0000000000	0.0000000000	0.0000000000	0.0001079331	0.0000000000	0.0000000000	0.0000000000	0.0000000000	0.0000000000	0.0000000000	0.0000000000	0.0000000000	0.0000000000	0.0000000000	0.0000000000	0.0000077095	no
	Bacteria;Firmicutes;Clostridia;Clostridiales;Veillonellaceae;Succiniclasticum	0.0000000000	0.0000000000	0.0000000000	0.0001360359	0.0000000000	0.0000000000	0.0000000000	0.0000000000	0.0000000000	0.0000000000	0.0000000000	0.0001245795	0.0000000000	0.0000000000	0.0000000000	0.0000000000	0.0000000000	0.0000000000	0.0000000000	0.0000000000	0.0000000000	0.0000000000	0.0000000000	0.0000000000	0.0000000000	0.0000000000	0.0000000000	0.0000000000	0.0000186154	0.0000000000	no
	Bacteria;Firmicutes;Clostridia;Clostridiales;Veillonellaceae;Veillonella	0.0000000000	0.0000000000	0.0000000000	0.0000000000	0.0000000000	0.0001546312	0.0000000000	0.0000000000	0.0000000000	0.0000000000	0.0000000000	0.0000000000	0.0000000000	0.0000000000	0.0000000000	0.0000000000	0.0000000000	0.0000000000	0.0000000000	0.0000000000	0.0002287544	0.0000000000	0.0000000000	0.0000000000	0.0000000000	0.0000000000	0.0000000000	0.0000000000	0.0000110451	0.0000163396	no
	Bacteria;Firmicutes;Clostridia;Other;Other;Other	0.0182433909	0.0262014483	0.0198438517	0.0323765474	0.0154954465	0.0106695531	0.0086450802	0.0212984347	0.0114320836	0.0208707671	0.0228638620	0.0348822723	0.0065856130	0.0195783133	0.0247307195	0.0193053847	0.0330356182	0.0234214787	0.0144881219	0.0377972466	0.0146402837	0.0165107320	0.0122360371	0.0190397351	0.0112570356	0.0175574205	0.0069814674	0.0056872038	0.0192133331	0.0183348918	no
	Bacteria;Firmicutes;Erysipelotrichi;Erysipelotrichales;Erysipelotrichaceae;Allobaculum	0.0000000000	0.0000000000	0.0000000000	0.0000000000	0.0000000000	0.0000000000	0.0000000000	0.0000000000	0.0000000000	0.0000000000	0.0000000000	0.0000000000	0.0000000000	0.0000000000	0.0000000000	0.0000000000	0.0000000000	0.0000000000	0.0000000000	0.0000000000	0.0000000000	0.0000000000	0.0000000000	0.0001034768	0.0000000000	0.0000000000	0.0000000000	0.0000000000	0.0000000000	0.0000073912	no
	Bacteria;Firmicutes;Erysipelotrichi;Erysipelotrichales;Erysipelotrichaceae;Other	0.0000000000	0.0000000000	0.0003253090	0.0000000000	0.0000000000	0.0000000000	0.0001571833	0.0000000000	0.0000000000	0.0000000000	0.0000000000	0.0000000000	0.0000000000	0.0000000000	0.0000000000	0.0000000000	0.0000000000	0.0000000000	0.0000000000	0.0000000000	0.0000000000	0.0000000000	0.0000000000	0.0000000000	0.0000000000	0.0000000000	0.0000000000	0.0000000000	0.0000344637	0.0000000000	no
	Bacteria;Firmicutes;Other;Other;Other;Other	0.0040419489	0.0013166557	0.0027651269	0.0019045028	0.0027184994	0.0026287305	0.0017290160	0.0115473441	0.0012292563	0.0023496890	0.0107133525	0.0074747726	0.0070921986	0.0038229842	0.0035329599	0.0047317119	0.0050575702	0.0047490556	0.0043677426	0.0110137672	0.0183003546	0.0027517887	0.0030590093	0.0017591060	0.0064944436	0.0016563604	0.0036811373	0.0017772512	0.0043810055	0.0052094470	no
	Bacteria;Other;Other;Other;Other;Other	0.0278566747	0.0430546412	0.0774235524	0.0157801660	0.0477096643	0.0432967373	0.0732474065	0.0382345394	0.0442532268	0.0514167243	0.0472955317	0.0459698518	0.0733282675	0.0370713624	0.0253339078	0.0348253998	0.0491767997	0.0113329736	0.0470863961	0.0165206508	0.0264211369	0.0434782609	0.0407538978	0.0494619205	0.1040554193	0.0456051237	0.0524244732	0.0251184834	0.0475670247	0.0408282031	no
	Bacteria;Proteobacteria;Alphaproteobacteria;Rhodobacterales;Rhodobacteraceae;Loktanella	0.0000000000	0.0000000000	0.0000000000	0.0000000000	0.0000000000	0.0000000000	0.0000000000	0.0000000000	0.0000000000	0.0000000000	0.0000000000	0.0000000000	0.0000000000	0.0000000000	0.0000000000	0.0000000000	0.0000000000	0.0000000000	0.0000000000	0.0000000000	0.0000000000	0.0000000000	0.0000986777	0.0000000000	0.0000000000	0.0000000000	0.0000000000	0.0000000000	0.0000000000	0.0000070484	no
	Bacteria;Proteobacteria;Alphaproteobacteria;Rhodospirillales;Acetobacteraceae;Craurococcus	0.0000000000	0.0000000000	0.0000000000	0.0000000000	0.0000000000	0.0000000000	0.0000000000	0.0000000000	0.0000000000	0.0000000000	0.0000000000	0.0000000000	0.0000000000	0.0000000000	0.0000000000	0.0000000000	0.0000000000	0.0000000000	0.0000000000	0.0000000000	0.0000000000	0.0000000000	0.0000986777	0.0000000000	0.0000000000	0.0000000000	0.0000000000	0.0000000000	0.0000000000	0.0000070484	no
	Bacteria;Proteobacteria;Betaproteobacteria;Burkholderiales;Alcaligenaceae;Parasutterella	0.0002184837	0.0000000000	0.0000000000	0.0000000000	0.0000000000	0.0001546312	0.0000000000	0.0000000000	0.0000000000	0.0000000000	0.0000000000	0.0000000000	0.0001266464	0.0000000000	0.0000000000	0.0000000000	0.0000000000	0.0001079331	0.0000000000	0.0000000000	0.0000000000	0.0000000000	0.0000000000	0.0000000000	0.0000000000	0.0000000000	0.0000000000	0.0000000000	0.0000356972	0.0000077095	no
	Bacteria;Proteobacteria;Deltaproteobacteria;Desulfovibrionales;Desulfohalobiaceae;Other	0.0000000000	0.0000000000	0.0000000000	0.0000000000	0.0000000000	0.0000000000	0.0000000000	0.0000000000	0.0000000000	0.0000000000	0.0000000000	0.0000000000	0.0000000000	0.0001158480	0.0000000000	0.0000000000	0.0000000000	0.0000000000	0.0000000000	0.0000000000	0.0000000000	0.0000000000	0.0000000000	0.0000000000	0.0000000000	0.0000000000	0.0000000000	0.0000000000	0.0000082749	0.0000000000	no
	Bacteria;Proteobacteria;Deltaproteobacteria;Desulfovibrionales;Desulfovibrionaceae;Other	0.0000000000	0.0000000000	0.0001626545	0.0789008298	0.0088351230	0.0214937374	0.0116315624	0.0003849115	0.0212661340	0.0012439530	0.0002613013	0.0085959885	0.0084853090	0.0005792400	0.0271434726	0.0192107504	0.0332508340	0.0063680518	0.0102269096	0.0002503129	0.0000000000	0.0000000000	0.0000000000	0.0000000000	0.0158753067	0.0394213781	0.0120588982	0.0000000000	0.0115600532	0.0117004224	no
	Bacteria;Proteobacteria;Deltaproteobacteria;Desulfovibrionales;Other;Other	0.0048066419	0.0084265964	0.0060182173	0.0066657598	0.0017670246	0.0015463120	0.0034580321	0.0079548371	0.0046711739	0.0222529371	0.0014371570	0.0026161704	0.0012664640	0.0018535681	0.0018957346	0.0017980505	0.0023673733	0.0124123044	0.0017044849	0.0086357947	0.0011437722	0.0095762245	0.0071047957	0.0038286424	0.0018761726	0.0025397527	0.0008885504	0.0005924171	0.0053386351	0.0040260050	no
	Bacteria;Proteobacteria;Deltaproteobacteria;Other;Other;Other	0.0001092419	0.0001316656	0.0001626545	0.0014963950	0.0000000000	0.0006185248	0.0004715498	0.0003849115	0.0001229256	0.0000000000	0.0001306506	0.0002491591	0.0002532928	0.0000000000	0.0002585093	0.0004731712	0.0003228236	0.0007555316	0.0002130606	0.0006257822	0.0000000000	0.0002201431	0.0000000000	0.0000000000	0.0007216048	0.0007729682	0.0013962935	0.0001184834	0.0002950694	0.0004198837	no
	Bacteria;Proteobacteria;Epsilonproteobacteria;Campylobacterales;Campylobacteraceae;Arcobacter	0.0000000000	0.0000000000	0.0000000000	0.0000000000	0.0000000000	0.0000000000	0.0000000000	0.0001283038	0.0000000000	0.0000000000	0.0000000000	0.0000000000	0.0000000000	0.0000000000	0.0000000000	0.0000000000	0.0000000000	0.0000000000	0.0000000000	0.0000000000	0.0000000000	0.0000000000	0.0000000000	0.0000000000	0.0000000000	0.0000000000	0.0000000000	0.0000000000	0.0000091646	0.0000000000	no
	Bacteria;Proteobacteria;Gammaproteobacteria;Enterobacteriales;Enterobacteriaceae;Escherichia/Shigella	0.0000000000	0.0000000000	0.0000000000	0.0000000000	0.0000000000	0.0000000000	0.0000000000	0.0000000000	0.0000000000	0.0000000000	0.0000000000	0.0000000000	0.0000000000	0.0000000000	0.0000000000	0.0000000000	0.0000000000	0.0000000000	0.0000000000	0.0001251564	0.0000000000	0.0000000000	0.0000000000	0.0000000000	0.0000000000	0.0000000000	0.0000000000	0.0000000000	0.0000000000	0.0000089397	no
	Bacteria;Proteobacteria;Gammaproteobacteria;Enterobacteriales;Enterobacteriaceae;Other	0.0000000000	0.0000000000	0.0000000000	0.0000000000	0.0000000000	0.0000000000	0.0000000000	0.0000000000	0.0000000000	0.0000000000	0.0000000000	0.0002491591	0.0000000000	0.0000000000	0.0000000000	0.0000000000	0.0000000000	0.0000000000	0.0000000000	0.0000000000	0.0000000000	0.0000000000	0.0000000000	0.0000000000	0.0000000000	0.0000000000	0.0000000000	0.0000000000	0.0000177971	0.0000000000	no
	Bacteria;Proteobacteria;Gammaproteobacteria;Other;Other;Other	0.0000000000	0.0000000000	0.0000000000	0.0000000000	0.0000000000	0.0000000000	0.0000000000	0.0000000000	0.0000000000	0.0000000000	0.0000000000	0.0000000000	0.0000000000	0.0000000000	0.0000000000	0.0000000000	0.0000000000	0.0000000000	0.0000000000	0.0000000000	0.0001143772	0.0000000000	0.0000000000	0.0000000000	0.0000000000	0.0000000000	0.0000000000	0.0000000000	0.0000000000	0.0000081698	no
	Bacteria;Proteobacteria;Other;Other;Other;Other	0.0000000000	0.0003949967	0.0000000000	0.0076180112	0.0012233247	0.0032472553	0.0011002829	0.0002566076	0.0028272895	0.0002764340	0.0000000000	0.0004983182	0.0005065856	0.0000000000	0.0028436019	0.0022712217	0.0045195308	0.0008634647	0.0015979546	0.0000000000	0.0000000000	0.0000000000	0.0000000000	0.0001034768	0.0014432097	0.0038648410	0.0021579081	0.0000000000	0.0012820790	0.0014046578	no
	Bacteria;TM7;TM7_genera_incertae_sedis;Other;Other;Other	0.0081931396	0.0038183015	0.0084580351	0.0042171133	0.0059806987	0.0018555745	0.0022005659	0.0107775212	0.0052858021	0.0033172080	0.0218186569	0.0042357045	0.0050658561	0.0020852641	0.0048255062	0.0108829374	0.0037662757	0.0079870480	0.0054330457	0.0025031289	0.0133821343	0.0001100715	0.0000986777	0.0000000000	0.0020204936	0.0007729682	0.0012693577	0.0000000000	0.0062363887	0.0037894032	no
	Bacteria;Tenericutes;Mollicutes;Anaeroplasmatales;Anaeroplasmataceae;Anaeroplasma	0.0007646930	0.0001316656	0.0004879636	0.0001360359	0.0000000000	0.0000000000	0.0000000000	0.0002566076	0.0000000000	0.0000000000	0.0003919519	0.0000000000	0.0000000000	0.0002316960	0.0010340371	0.0008517081	0.0002152158	0.0000000000	0.0000000000	0.0003754693	0.0074345190	0.0001100715	0.0001973554	0.0000000000	0.0001443210	0.0003312721	0.0007616146	0.0000000000	0.0001714724	0.0008182560	no
	Unclassified;Other;Other;Other;Other;Other	0.0000000000	0.0000000000	0.0000000000	0.0000000000	0.0000000000	0.0000000000	0.0000000000	0.0000000000	0.0000000000	0.0000000000	0.0000000000	0.0001245795	0.0000000000	0.0000000000	0.0000000000	0.0000000000	0.0000000000	0.0000000000	0.0000000000	0.0000000000	0.0000000000	0.0000000000	0.0000000000	0.0000986777	0.0000000000	0.0000000000	0.0000000000	0.0000000000	0.0000088985	0.0000070484	no
